# Caspase-11 mediated inflammasome activation in macrophages by systemic infection of *A. actinomycetemcomitans* exacerbates arthritis

**DOI:** 10.1038/s41368-024-00315-x

**Published:** 2024-08-15

**Authors:** Tokuju Okano, Hiroshi Ashida, Noriko Komatsu, Masayuki Tsukasaki, Tamako Iida, Marie Iwasawa, Yuto Takahashi, Yasuo Takeuchi, Takanori Iwata, Miwa Sasai, Masahiro Yamamoto, Hiroshi Takayanagi, Toshihiko Suzuki

**Affiliations:** 1 Department of Bacterial Pathogenesis, Infection and Host Response, Graduate School of Medical and Dental Sciences, Tokyo Medical and Dental University, Tokyo, Japan; 2 Department of Immunology, Graduate School of Medicine and Faculty of Medicine, The University of Tokyo, Tokyo, Japan; 3 Department of Osteoimmunology, Graduate School of Medicine and Faculty of Medicine, The University of Tokyo, Tokyo, Japan; 4 Department of Lifetime Oral Health Care Science, Graduate School of Medical and Dental Sciences, Tokyo Medical and Dental University, Tokyo, Japan; 5 Department of Periodontology, Graduate School of Medical and Dental Sciences, Tokyo Medical and Dental University, Tokyo, Japan; 6 Department of Immunoparasitology, Research Institute for Microbial Diseases, Osaka University, Osaka, Japan

**Keywords:** Bacterial pathogenesis, Cellular microbiology, Mechanisms of disease

## Abstract

Clinical studies have shown that *Aggregatibacter actinomycetemcomitans* (*A. actinomycetemcomitans*) is associated with aggressive periodontitis and can potentially trigger or exacerbate rheumatoid arthritis (RA). However, the mechanism is poorly understood. Here, we show that systemic infection with *A. actinomycetemcomitans* triggers the progression of arthritis in mice anti-collagen antibody-induced arthritis (CAIA) model following IL-1β secretion and cell infiltration in paws in a manner that is dependent on caspase-11-mediated inflammasome activation in macrophages. The administration of polymyxin B (PMB), chloroquine, and anti-CD11b antibody suppressed inflammasome activation in macrophages and arthritis in mice, suggesting that the recognition of lipopolysaccharide (LPS) in the cytosol after bacterial degradation by lysosomes and invasion via CD11b are needed to trigger arthritis following inflammasome activation in macrophages. These data reveal that the inhibition of caspase-11-mediated inflammasome activation potentiates aggravation of RA induced by infection with *A. actinomycetemcomitans*. This work highlights how RA can be progressed by inflammasome activation as a result of periodontitis-associated bacterial infection and discusses the mechanism of inflammasome activation in response to infection with *A*. *actinomycetemcomitans*.

## Introduction

Periodontitis is a chronic inflammatory disease caused by bacterial infection in the periodontium that leads to the secretion of numerous proinflammatory cytokines, such as IL-1β, TNFα, and IL-6, from gingival tissue or immune cells, resulting in the expansion of the inflammatory response systemically.^1–5^ Dental biofilms or plaques are required to induce periodontitis; however, recent studies based on independent metagenomic and mechanistic approaches have hypothesized that the pathogenesis of periodontitis includes polymicrobial synergy and the dysbiosis of periodontitis-associated bacteria.^6^ The dysbiosis of periodontal microbiota results a change in the relative abundance in health, leading to alterations in host-microbe crosstalk sufficient to mediate destructive inflammation and bone loss.^7^ Both the inflammatory response induced by periodontitis-associated bacteria and host-microbe crosstalk arising from polymicrobial synergy and dysbiosis are considered to play important roles in the pathogenesis of periodontitis. Periodontitis-associated bacteria are classified by their pathogenicity and properties into closely interrelated complexes. Especially, the highly pathogenic bacteria comprising the ‘red complex’ (*Porphyromonas gingivalis*, *Treponema denticola*, and *Tannerella forsythia*) are important in adult periodontitis, while *Aggregatibacter actinomycetemcomitans* can be a key factor in aggressive or juvenile periodontitis.^1,8,9^ Previous reports have shown that the spread of inflammation arising from periodontitis or periodontitis-associated bacterial infection can induce autoimmune diseases, including rheumatoid arthritis (RA), multiple sclerosis, infective endocarditis and ulcerative colitis; however, the critical molecular mechanisms are poorly understood.^10–13^

RA is a heterogenous chronic inflammatory autoimmune disease with the hallmarks of synovial inflammation and autoantibody production, leading to bone destruction. The activation of Toll-like receptors (TLRs) signaling and (NOD)-like receptor (NLR) signaling in a variety of cells, such as macrophages, dendritic cells, T cells and B cells, contributes to the chronic course of RA. The activation of these signaling pathways can be induced by microbial-associated molecular patterns (MAMPs), such as lipopolysaccharide (LPS) and peptidoglycan (PGN) expressed in microorganisms, or danger-associated molecular patterns (DAMPs),^14^ such as reactive oxygen species and ER stress generated in the cytosol. The activation of these signaling pathways also guides the secretion of proinflammatory cytokines and chemokines such as IL-1β, TNFα, IL-6, IL-8, and Type I interferons (IFNs), resulting in T-cell differentiation and activation, B cell activation, and autoantibody production. Autoantibodies against post-translationally modified protein antigens are necessary for RA to be triggered. A major example is anti-citrullinated protein antibodies (ACPAs). One report demonstrated that *A. actinomycetemcomitans* a common virulence factor, leukotoxin A (LtxA), produced by *A. actinomycetemcomitans* contributes to the generation of autoantigens that trigger RA, resulting in the progression of RA induced by cellular hypercitrullination in neutrophils; this previous report provided clinical data supporting a link between periodontal infection and RA.^15^ Other reports showed the similarity of periodontal or hematological characteristics such as cytokine production in blood between aggressive periodontitis and rheumatoid arthritis.^16,17^ Moreover, some clinical reports demonstrated that *A. actinomycetemcomitans*, *P. gingivalis* and *T. forsythia* were detected in peripheral blood from patients or healthy donor,^18,19^ suggesting the possibility that oral bacteria can spread from blood to localized places or oral to blood.

RA and periodontitis have much relation with inflammasome consists of a complex of caspase-1, nucleotide- binding oligomerization domain NLR family, and the adaptor protein apoptosis-associated speck-like protein containing a caspase recruitment domain (ASC) regulates caspase-1 cleavage and IL-1β secretion from cells by recognition of MAMPs or DAMPs.^14,20–26^ The inflammasome also controls pyroptosis, which is a cell death pathway executed by gasdermin D (GSDMD) in a manner that is dependent on cleaved caspase-1 and caspase-11.^27,28^ Recent studies have revealed that *A. actinomycetemcomitans* activates inflammasome NLR family pyrin domain containing 3 (NLRP3) or absent in melanoma 2 (AIM2) dependent manner in human macrophage by using small interference RNA analysis, and controls cell death through caspase-11 related pathway in murine macrophage by analysis of gene expression level, and murine alveolar bone loss by injected *A. actinomycetemcomitans* to gingival tissue was attenuated in NLRP3 or caspase-11-deficient mice, but relationships with pathogenesis for periodontitis or autoimmune diseases are elusive.^29–32^ In the present study, we demonstrated that LPS from *A. actinomycetemcomitans* activates the inflammasome in a caspase-11-dependent manner that is mediated by binding with CD11b and lysosomal degradation. Furthermore, we also discovered that inflammasome activation by infection with *A. actinomycetemcomitans* triggers progression of RA in a mice CAIA model.

## Results

### Infection with *A. actinomycetemcomitans* exacertbates arthritis in a CAIA model in a manner that is dependent on IL-1β secretion by phagocytic cells

**Fig. 1:**
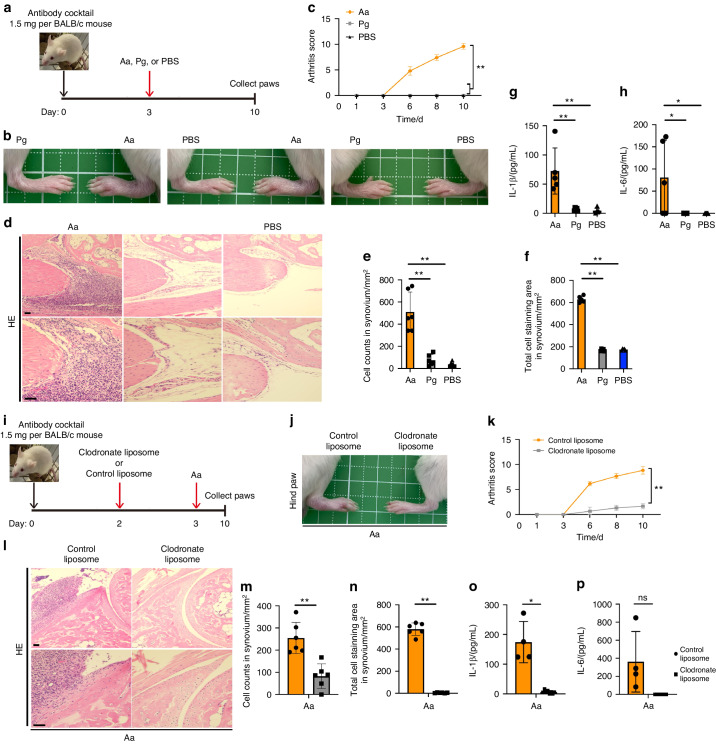
*A*. *actinomycetemcomitans* exacerbates arthritis in a CAIA model in a macrophage-dependent manner. **a**–**h** BALB/c mice were intraperitoneally injected with 1.5 mg of anti-collagen antibody on day 0 and with *A. actinomycetemcomitans* ATCC29522 (Aa), *P. gingivalis* ATCC33277 (Pg) or PBS on day 3. The arthritis scores were then monitored for 10 days. All paws were collected on day 10 for HE staining or cytokine analysis (n = 5 mice per group). **a** Schematic of CAIA model infected with *A. actinomycetemcomitans* or *P. gingivalis*. **b** Representative photographs of hind paws. **c** Arthritis score. **d** Representative HE staining of hind paws. Scale bar, 100 μm. **e** Cell number in 1 mm^2^ synovium. **f** Stained cell area in 1 mm^2^ synovium. **g**, **h** ELISA analysis for IL-1β and IL-6 release in paws **i**–**p** BALB/c mice were intraperitoneally injected with 1.5 mg of anti-collagen antibody on day 0, followed by the injection of liposomes containing clodronate or PBS on day 2 and *A. actinomycetemcomitans* (Aa) on day 3. The arthritis scores were monitored for 10 days. The paws of all the mice were collected on day 10 for HE staining or cytokine analysis (clodronate liposome, *n* = 8; control liposome, *n* = 4). **g** Schematic of CAIA model infected with *A. actinomycetemcomitans* and administered 10 μL/g of liposomes containing clodronate or PBS. Scale bar, 100 μm. **h** Representative photographs of hind paws. **i** Arthritis score. **j** Representative HE staining of hind paws. **k**, **l** ELISA analysis showing the release of IL-1β and IL-6 in paws. **c**, **k** ^∗∗^*P* < 0.01 indicates a statistically significant difference using a one-way ANOVA with the Tukey test (ns, not significant). **e**–**h**, **m**–**p** ^∗^*P* < 0.05 and ^∗∗^*P* < 0.01 indicate a statistically significant difference using a *t*-test (ns, not significant)

Previous reports have shown that systemic infection with periodontitis-associated bacteria potentiates the induction of RA through the spread of inflammatory cytokines, such as IL-1β and IL-6.^33,34^ To examine how infection with *A. actinomycetemcomitans* can trigger arthritis systemically, we used a CAIA model in BALB/c mice infected with *A. actinomycetemcomitans* ATCC29522 isolated from mandibular abscess. We established scheme of CAIA model for *A. actinomycetemcomitans* infection to BALB/c mice (Fig. 1a). Infection with *P. gingivalis* ATCC33277 and the administration of PBS were used as negative controls. Intraperitoneal infection with *A. actinomycetemcomitans*, but not infection with *P. gingivalis* ATCC33277 or PBS administration, induced paw swelling (Fig. 1b). The arthritis severity was higher in the *A. actinomycetemcomitans*-infected CAIA model than in the *P. gingivalis*-infected or PBS-administered groups (Fig. 1c). Hematoxylin and eosin (HE) staining showed cell infiltration in the paws of the *A. actinomycetemcomitans*-infected group (Fig. 1d). We also counted cells and measured cell staining area in 1 mm^2^ synovium. We detected the highest number and area in *A. actinomycetemcomitans*-infected group (Fig. 1e, f). The secretion of IL-1β and IL-6 was detected in the paws of the *A. actinomycetemcomitans*-infected group but not in those of mice infected with *P. gingivalis* or administered with PBS (Fig. 1g, h). The CAIA model is characterized by the activations of neutrophils and macrophages, but not of B lymphocytes or T lymphocytes.^11,35^ To assess the function of phagocytic cells in the CAIA model with using LPS from *E. coli* (LPS EC) as a general booster of this model, we administered liposomes containing clodronate, which cause the systemic depletion of major phagocytic cells, such as macrophages (Fig. S1a).^36,37^ Paw swelling and the arthritis severity were diminished by clodronate treatment (Fig. S1b and S1c). IL-1β but not IL-6 production in paws were suppressed by clodronate treatment (Fig. S1d and S1e). These data provide that macrophages have critical role to aggravate arthritis in the CAIA model. To evaluate the aggravation of arthritis by *A. actinomycetemcomitans*-infection is dependent on macrophages, we administered liposomes containing clodronate and infected *A. actinomycetemcomitans* to mice (Fig. 1i). Paw swelling and arthritis severity in the *A. actinomycetemcomitans*-infected CAIA mice were attenuated by the administration of clodronate-containing liposomes but not liposomes containing PBS as a negative control (Fig. 1j, k). HE staining showed cell infiltration in the paws of the PBS-containing liposomes administration group but not clodronate-containing liposomes administration group in *A. actinomycetemcomitans*-infected CAIA model (Fig. 1l). We also counted cell number and measured cell staining area in 1 mm^2^ synovium. The administration of clodronate-containing liposomes reduced cell number and staining area in synovium (Fig. 1m, n). IL-1β secretion in the paws of mice administered with clodronate-containing liposomes was also significantly reduced, compared with that in mice treated with PBS-containing liposomes (Fig. 1o). However, IL-6 secretion in the paws of mice was not affected by clodronate-containing liposomes (Fig. 1p). Together, these findings indicate that the release of IL-1β, but not ΙL-6, from phagocytic cells such as macrophages is an important factor in the induction of arthritis in response to infection with *A. actinomycetemcomitans*.

### NLRP3 is essential to activate inflammasome in macrophages by *A. actinomycetemcomitans* infection in a LtxA-independent manner

**Fig. 2:**
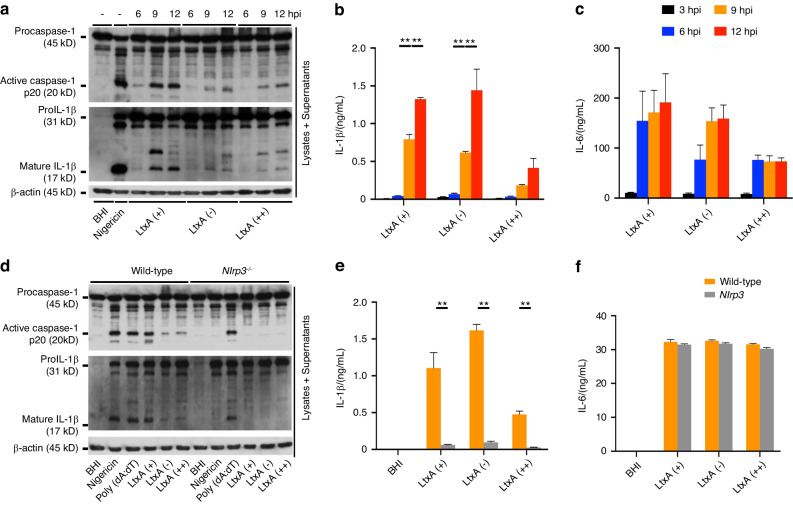
NLRP3 inflammasome is activated by infection with *A*. *actinomycetemcomitans*. BMDMs from wild-type or NLRP3-deficient (*Nlrp3*^*−/−*^) mice were infected with *A. actinomycetemcomitans* LtxA (+) (ATCC29522), LtxA (-) (ATCC43717), or LtxA (++) (JP2). Then, 5 μmol/L of nigericin or 2 μg of Poly (dA:dT) were administered together with 200 ng of LPS-EB-primed BMDMs as a control to activate NLRP3 or AIM2 inflammasomes. Brain heart infusion (BHI) media was added as a negative control for inflammasome activation. Cell lysates and culture supernatants were harvested at (**a**–**c**) the indicated times (hpi, h post-infection) or (**d**–**f**) 9 hpi. **a**, **d** Immunoblot analysis for procaspase-1, pro-IL-1β, and β-actin in cells and active caspase-1 and mature IL-1β in supernatants. **b**, **c**, **e**, **f** ELISA analysis for IL-1β and IL-6 release in supernatants. **a**, **d** Blots are representative of three independent experiments. **b**, **c**, **e**, **f** Data are shown as the mean ± SD of triplicates and are representative of three independent experiments. ^∗∗^*P* < 0.01 indicate a statistically significant difference using a *t*-test

To confirm that infection with *A. actinomycetemcomitans* triggers the release of IL-1β from macrophages, we checked the activation of inflammasomes in macrophages, which regulate IL-1β maturation following caspase-1 activation in response to infection with *A. actinomycetemcomitans*. Since several previous reports have shown that LtxA from *A. actinomycetemcomitans* is an important factor in the induction of the host immune response and cell death,^38–40^ we prepared 3 types of *A. actinomycetemcomitans* strains: ATCC29522 (non-JP2 genotype serotype b, LtxA positive, LtxA (+)), ATCC43717 (serotype a, LtxA negative, LtxA (-)), and JP2 (JP2 genotype serotype b, high expression of LtxA, LtxA (++)).^41,42^ JP2 genotype is the specific sequence to enhance the LtxA gene expression level.^42,43^ Bone marrow-derived macrophages (BMDMs) from C57BL/6 mice were infected with *A. actinomycetemcomitans* or LPS-primed BMDMs were administered nigericin as a control for NLRP3 inflammasome activation. Caspase-1 activation, IL-1β maturation, and IL-6 release were observed in infected BMDMs and administered with nigericin (Fig. 2a–c). Furthermore, to confirm that NLRP3 or AIM2 is important for inflammasome activation during infection with *A. actinomycetemcomitans*, as reported in previous studies, we used BMDMs from wild-type, NLRP3-deficient, or AIM2-deficient mice. Poly (dA:dT) was used as a control for AIM2 inflammasome activation. Caspase-1 activation and IL-1β maturation were observed in BMDMs from AIM2-deficient mice and wild-type mice infected with *A. actinomycetemcomitans*, but not in BMDMs from NLRP3-deficient mice infected with *A. actinomycetemcomitans* (Figs. S2a and S2b, 2d, e). IL-6 release was detected in BMDMs from wild-type and NLRP3-deficient mice (Fig. 2f). These results suggest that NLRP3, but not AIM2, is necessary for inflammasome activation in macrophages during infection with *A. actinomycetemcomitans*.

### Caspase-11 triggers noncanonical NLRP3-mediated inflammasome activation in macrophages during infection with *A. actinomycetemcomitans*

**Fig. 3:**
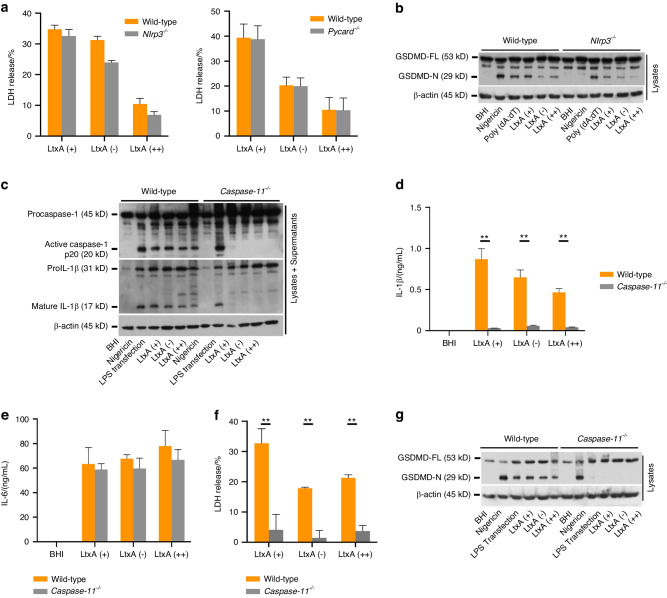
Caspase-11-mediated NLRP3 inflammasome is activated by infection with *A*. *actinomycetemcomitans*. BMDMs from wild-type, NLRP3-deficient (*Nlrp3*^*−/−*^), ASC-deficient (*Pycard*^*−/−*^) or caspase-11-deficient (*Caspase-11*^*−/−*^) mice were infected with *A. actinomycetemcomitans* LtxA (+) (ATCC29522), LtxA (-) (ATCC43717), or LtxA (++) (JP2). Nigericin (5 μmol/L) or Poly (dA:dT) (2 μg) were administered to LPS-EB-primed BMDMs as a control to activate NLRP3 or AIM2 inflammasomes, or 5 μg of LPS-EB was transfected into BMDMs as a control to activate caspase-11-mediated non-canonical NLRP3 inflammasomes. BHI media was added as a negative control for inflammasome activation. Cell lysates and culture supernatants were harvested at 9 hpi. **a** and **f** LDH release assay in culture supernatants. **b**, **g** Immunoblot analysis for full-length GSDMD (GSDMD-FL, 55 kDa) N terminal of GSDMD (GSDMD-N, 30 kDa), or β-actin in cells. **c** Immunoblot analysis for procaspase-1, pro-IL-1β, and β-actin in cells and active caspase-1 and mature IL-1β in supernatants. **d**, **e** ELISA analysis for IL-1β and IL-6 release in supernatants. **b**, **c**, **g** Blots are representative of three independent experiments. **a**, **d**, **e**, **f** Data are shown as the mean ± SD of triplicates and are representative of three independent experiments. ^∗∗^*P* < 0.01 indicate a statistically significant difference using a *t*-test

When we examined cell morphology, cell swelling was observed in BMDMs from not only wild-type mice, but also NLRP3-deficient mice infected with *A. actinomycetemcomitans* (data not shown). To assess the cytotoxicity of infection with *A. actinomycetemcomitans* in the absence of inflammasome activation in BMDMs, we examined LDH release from BMDMs. No significant differences in LDH release by BMDMs were observed when BMDMs from wild-type, NLRP3-deficient, and ASC *(Pycard*^*−/−*^)-deficient mice were compared (Fig. 3a, b). We postulated that the cell death by *A. actinomycetemcomitans* is mediated by GSDMD which is converted to its pore-forming form (N terminal GSDMD, an execution factor for pyroptosis) in the cell membrane at a stage upstream of inflammasome activation.^27,28^ Actually, we observed GSDMD cleavage in BMDMs from NLRP3-deficient mice infected with *A. actinomycetemcomitans* (Fig. 3c). These results suggested that NLRP3-mediated inflammasome activation during infection with *A. actinomycetemcomitans* is triggered after the induction of pyroptosis by GSDMD. GSDMD is cleaved by caspase-11 in a step that occurs upstream of caspase-1 activation and causes potassium ion efflux, resulting in non-canonical NLRP3-mediated inflammasome activation. To determine whether infection with *A. actinomycetemcomitans* activates non-canonical NLRP3-mediated inflammasomes, we examined BMDMs from caspase-11-deficient mice. Caspase-1 activation and IL-1β release by *A. actinomycetemcomitans* infection were not observed in BMDMs from caspase-11-deficient mice, whilst IL-6 release was not significantly difference in BMDMs from between wild-type and caspase-11-deficient mice (Fig. 3d, e). LDH release and GSDMD cleavage were also diminished in BMDMs from caspase-11-deficient mice (Fig. 3f, g). Additionally, we used JCM30399 strain isolated from the periodontal pocket as a clinically isolated strain. LDH and IL-1β release were diminished in BMDMs from caspase-11-deficient mice compared to that from wild-type mice (Fig. S3a, b). These results suggest that infection with *A. actinomycetemcomitans* triggers noncanonical NLRP3-mediated inflammasome activation through caspase-11.

### LPS from *A. actinomycetemcomitans* directly activates inflammasomes

**Fig. 4:**
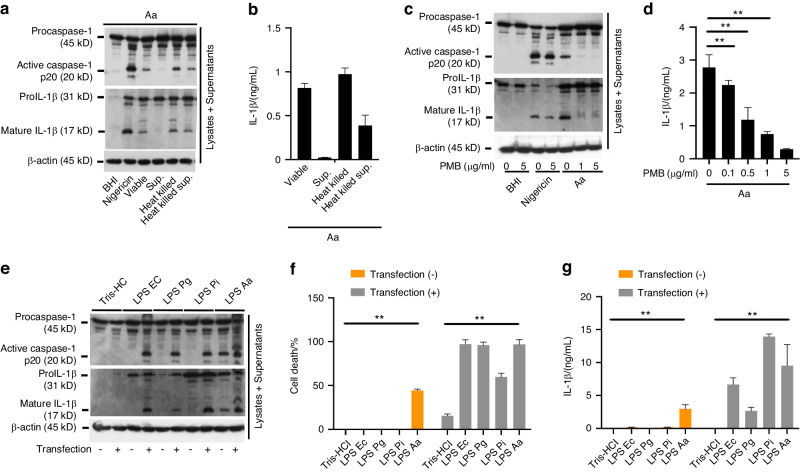
LPS from *A*. *actinomycetemcomitans* triggers inflammasome activation. **a**, **b** *A. actinomycetemcomitans* ATCC29522 (Viable) was infected or bacterial culture supernatants (Sup.), heat-killed (95 °C) bacterial culture (Heat killed), or supernatants from heat-killed bacterial culture (Heat killed sup.) were added to BMDMs for 9 h. Nigericin (5 μmol/L) was administered to the BMDMs as a control to activate NLRP3 inflammasomes. BHI media was added as a negative control for inflammasome activation. Cell lysates and culture supernatants were harvested at 9 hpi. **c**, **d** PMB (0, 1, or 5 μg/mL)-administered BMDMs were infected with *A. actinomycetemcomitans* ATCC29522 (Aa) for 9 h. Cell lysates and culture supernatants were harvested at 9 hpi. Nigericin (5 μmol/L) was administered to BMDMs as a control to activate NLRP3 inflammasomes. BHI media was added as a negative control for inflammasome activation. **e**–**g** BMDMs were subjected to 10 μg of LPS from *E. coli* MC1061 (LPS Ec), *P. gingivalis* ATCC33277 (LPS Pg), *P. intermedia* ATCC25611 (LPS Pi) or LPS from *A. actinomycetemcomitans* ATCC29522 (LPS Aa) with or without transfection for 10 h. Nigericin (5 μmol/L) was administered to BMDMs as a control to activate NLRP3 inflammasomes. Tris-HCl was added as a negative control for inflammasome activation. Cell lysates and culture supernatants were harvested at 9 h post-addition. **a**, **c**, **e** Immunoblot analysis for procaspase-1, pro-IL-1β and β-actin in cells and active caspase-1 and mature IL-1β in supernatants. **b**, **d**, **g** ELISA analysis for IL-1β release in supernatants. **f** LDH release assay in culture supernatants. **a**, **c**, **e** Blots are representative of three independent experiments. **b**, **d**, **f**, **g** Data are shown as the mean ± SD of triplicates and are representative of three independent experiments. ^∗∗^*P* < 0.01 indicate a statistically significant difference using a *t*-test

We next sought to identify the bacterial component that triggers inflammasome activation during infection with *A. actinomycetemcomitans*. A recent study elucidated that LPS or outer membrane vesicles (OMV) from gram-negative bacteria activates noncanonical NLRP3-mediated inflammasomes through caspase-11 and lipoteichoic acid (LTA), while OMV from gram-positive bacteria activates noncanonical NLRP6-mediated inflammasomes through caspase-11.^44–48^ First, the bacterial culture of *A. actinomycetemcomitans* ATCC29522 has been fractionated and treated by heat treatment. The resultant four fractions, bacterial culture supernatants, heat-inactivated bacteria (incubated at 65 °C for 30 minutes), heat-killed bacteria (incubated at 100 °C for 30 min), or culture supernatants from heat-killed bacteria, were examined their ability to induce inflammasome activation. Caspase-1 activation and IL-1β cleavage/release were observed in BMDMs treated by heat-inactivated bacteria, heat-killed bacteria, and culture supernatants from heat-killed bacteria but not by bacterial culture supernatants (Fig. 4a, b). Since the bioactivities of proteins other than LPS are inactivated by heat-killed treatment, the results suggest that LPS from *A. actinomycetemcomitans* activates noncanonical NLRP3-mediated inflammasomes through caspase-11, and that this activation is not dependent on bacterial viability. To confirm the LPS-mediated inflammasome activation, we used PMB to inactivate LPS activity by binding to lipid A component. The co-incubation of *A. actinomycetemcomitans* with PMB did not affect the bacterial viability (Fig. S4a). Caspase-1 activation and IL-1β cleavage/release were attenuated by the addition of PMB in a dose-dependent manner (Fig. 4c, d). Furthermore, to confirm that pure LPS from *A. actinomycetemcomitans*, without the presence of other bacterial components, is sufficient to activate inflammasomes, we purified LPS from *A. actinomycetemcomitans* ATCC29522 and administered it with or without transfection to BMDMs. The cell death, caspase-1 activation, and IL-1β cleavage/release were observed by addition of LPS (10 μg) from *A. actinomycetemcomitans* with or without transfection. However, LPS (10 μg) from *Escherichia Coli* MC1061, *P. gingivalis* ATCC33277, or *Prevotella intermedia* ATCC25611 not without but with transfection induced inflammasome activation (Fig. 4e-g). These results suggest that LPS from *A. actinomycetemcomitans* has an ability to induce inflammasome activation simply by adding it to BMDMs. In contrast, IL-6 secretion in BMDMs subjected to LPS from *A. actinomycetemcomitans* was not different from that in BMDMs stimulated with LPS from other type of bacteria. We also examined the effect of a low concentration of LPS from *A. actinomycetemcomitans* on the secretion of IL-6, TNFα, and interferon β (IFNβ), which is important factor to express caspase-11. The release of IL-6, TNFα, and IFNβ from BMDMs stimulated with LPS from *A. actinomycetemcomitans* are same tendency compared to those stimulated with the LPS from *E. coli*, but not *P. gingivalis* and *P. intermedia* (Fig. S4b–d).

### Lysosomal degradation of bacteria and lysosomal disruption contribute to activate inflammasome

**Fig. 5:**
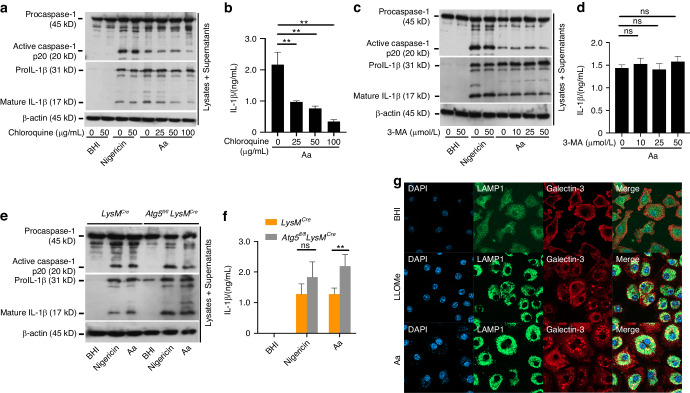
Chloroquine attenuates inflammasome activation induced by infection with *A*. *actinomycetemcomitans*. **a**–**d** Chloroquine (25, 50, or 100 μg/mL)-administered or 3-MA (10, 25, or 50 μg/mL)-administered BMDMs were infected with *A. actinomycetemcomitans* ATCC29522 (Aa) for 9 h. Cell lysates and culture supernatants were harvested at 9 hpi. Nigericin (5 μmol/L) was administered to BMDMs as a control to activate NLRP3 inflammasomes. **a**, **c** Immunoblot analysis for procaspase-1, pro-IL-1β and β-actin in cells and active caspase-1 and mature IL-1β in supernatants. **b**, **d** ELISA analysis for IL-1β release in supernatants. **e**, **f** BMDMs from *LysM-Cre* transgenic line or myeloid cell-specific Atg5-deficient (Atg5^*fl/fl*^*LysM*^*Cre*^) mice were infected with *A. actinomycetemcomitans* ATCC29522 (Aa). Nigericin (5 μmol/L) was administered to 200 ng of LPS-EB-primed BMDMs as a control to activate NLRP3 inflammasomes. BHI media was added as a negative control for inflammasome activation. Cell lysates and culture supernatants were harvested at 9 hpi. **e** Immunoblot analysis for procaspase-1, pro-IL-1β and β-actin in cells and active caspase-1 and mature IL-1β in supernatants. **f** ELISA analysis for IL-1β release in supernatants. **g** BMDMs were infected with *A. actinomycetemcomitans* ATCC29522 (Aa) for 4 h. Representative immunostaining analysis for lysosomal disruption (arrowheads indicate merge region). Scale bar, 20 μm. Nuclear was stained by DAPI, LAMP1 was stained using Alexa fluor 488, while galectin-3 was stained using Cy5. **a**, **c**, **e** Blots are representative of three independent experiments. **b**, **d**, **f** Data are shown as the mean ± SD of triplicates and are representative of three independent experiments. ^∗∗^*P* < 0.01 indicate a statistically significant difference using a *t*-test (ns not significant)

Next, we wondered how LPS from *A. actinomycetemcomitans* is recognized by caspase-11, and we assumed the phagocytic degradation of bacteria by macrophages to be an essential step. To confirm this hypothesis, we focused on lysosomal degradation with internal low pH in macrophages and examined the effect of chloroquine, an inhibitor to suppress fusion between phagosome and lysosome.^49,50^ As a first step, we assessed internal low pH of lysosome in BMDMs with *A. actinomycetemcomitans* infection and detected fluorescence intensity and observed promotion of internal low pH and higher fluorescence intensity in macrophages with *A. actinomycetemcomitans* ATCC29522 infection (Fig. S5a, b). Next, we used chloroquine and observed that caspase-1 activation and IL-1β cleavage/release *A. actinomycetemcomitan* infection were attenuated in BMDMs administered with chloroquine (Fig. 5a, b). However, chloroquine did not affect IL-6 release from BMDMs (Data not shown). We also examined whether chloroquine prevents inflammasome activation induced by nigericin, LPS from *E. coli*, heat-killed *A. actinomycetemcomitans*, or LPS from *A. actinomycetemcomitans*. IL-1β release from BMDMs was not altered significantly by chloroquine in the stimulation with any of the above factors (Fig. S5c), indicating that inflammasome activation by released LPS from *A. actinomycetemcomitans* is not affected by chloroquine. We observed that the growth of *A. actinomycetemcomitans* was not affected by chloroquine administration (Fig. S5d). In addition, to examine whether autophagosome recruitment is an essential step, we used 3-methyladenine (3-MA) to inhibit autophagy by blocking autophagosome formation via the inhibition of class III phosphatidylinositol 3-kinases (PI3K).^51^ Caspase-1 activation and IL-1β release were observed in BMDMs administered 3-MA as well as in BMDMs without 3-MA infected with *A. actinomycetemcomitans* (Fig. 5c, d). We also examined inflammasome activation using myeloid cell-specific autophagy-related gene 5 (Atg5)-deficient mice created by crossing *Atg5*^*fl/fl*^ mice with *LysM-Cre* transgenic mice (*Atg5* ^*fl/fl*^*LysM*^*Cre*^) that cannot induce autophagosome formation with light chain 3 (LC3).^52^ Caspase-1 activation and IL-1β release in BMDMs were not decreased, compared with that in BMDMs from wild-type mice (Fig. 5e, f). Next, to investigate how to be exposed to LPS from Aa in cytosol, we checked lysosomal disruption by observing colocalization of galectin-3 with lysosome-associated membrane proteins 1 (LAMP1) by *A. actinomycetemcomitans* infection.^53^ Colocalization of galectin-3 with LAMP1 was strongly detected in BMDMs with *A. actinomycetemcomitans* infection and L-Leucyl-L-Leucine methyl ester (LLOMe) treatment (Fig. 5g). This data indicates that *A. actinomycetemcomitans* induces lysosomal disruption and enhances the delivery of LPS from *A. actinomycetemcomitans* to cytosol. Together, these results suggest that lysosomal degradation without autophagosome formation and lysosomal disruption cause the release of LPS and activation of inflammasomes during infection with *A. actinomycetemcomitans*.

### CD11b is essential for bacterial internalization and inflammasome activation during *A. actinomycetemcomitans* infection

**Fig. 6:**
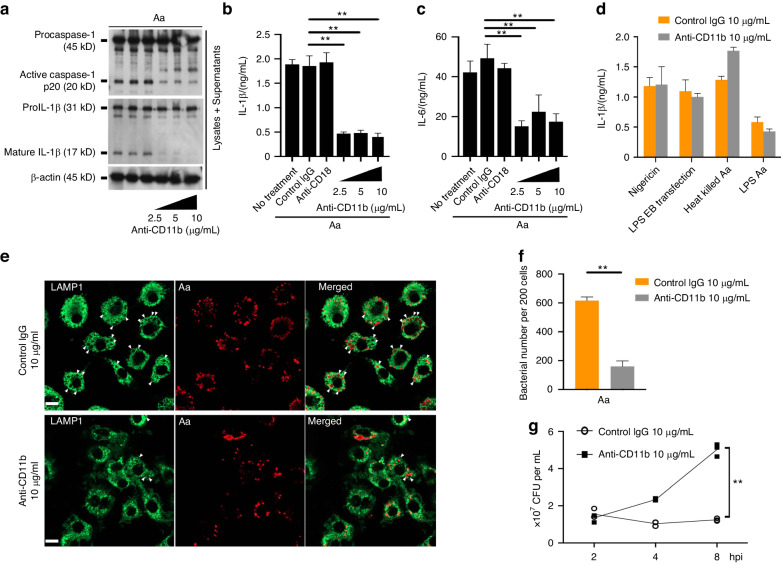
CD11b-mediated invasion to macrophages is an essential step in inflammasome activation in response to infection with *A*. *actinomycetemcomitans*. **a**–**c** Anti-CD11b antibody (2.5, 5, or 10 μg/mL)-administered, anti-CD18 antibody (10 μg/mL)-administered, or isotype control IgG (10 μg/mL)-administered BMDMs were infected with *A. actinomycetemcomitans* ATCC29522 (Aa) for 9 h. Cell lysates and culture supernatants were harvested at 9 hpi. **a** Immunoblot analysis for procaspase-1, pro-IL-1β and β-actin in cells and active caspase-1 and mature IL-1β in supernatants. **b**, **c** ELISA analysis for IL-1β and IL-6 release in supernatants. **d** Anti-CD11b antibody (10 μg/mL)-administered or isotype control IgG (10 μg/mL)-administered BMDMs were added or transfected with 10 μg of LPS-EB, 5 μmol/L of nigericin, heat-killed *A. actinomycetemcomitans* ATCC29522 (Heat killed Aa), or 10 μg of LPS from *A. actinomycetemcomitans* ATCC 29522 (LPS Aa). ELISA analysis for IL-1β release in supernatants. **e**, **f** Anti-CD11b antibody (10 μg/mL)-administered or isotype control IgG (10 μg/mL)-administered BMDMs were infected with *A. actinomycetemcomitans* ATCC29522 (Aa) for 4 h. **e** Representative immunostaining analysis for lysosomal formation (arrowheads indicate phagosome). Scale bar, 10 μm. LAMP1 was stained using Alexa fluor 488, while *A. actinomycetemcomitans* ATCC29522 was stained using Cy5. **f** Bacterial number in cytosol of BMDMs. **g** Anti-CD11b antibody (10 μg/mL)-administered or isotype control IgG (10 μg/mL)-administered BMDMs were infected with *A. actinomycetemcomitans* ATCC29522 (Aa) for the indicated time. Colony forming units (CFUs) in supernatants. **a** Blots are representative of three independent experiments. **b**–**d**, **f**, **g** Data are shown as the mean ± SD of triplicates and are representative of three independent experiments. ^∗∗^*P* < 0.01 indicate a statistically significant difference using a *t*-test. (**e**) Images are representative of three independent experiments

We next sought to identify the receptors necessary for the internalization of *A. actinomycetemcomitans* into the macrophages and the activation of inflammasomes. Myeloid cells as well as macrophages contain molecules such as integrins that are localized in the cell membrane for adhesion to traffic into tissues.^54^ A previous report showed that one of the β_2_ integrin family MAC-1 consisting of heterodimer of CD11b (α_M_) and CD18 (β_2_), or LFA-1, a heterodimer of CD11c (α_L_) and CD18 (β_2_) the molecule for adhesion, is essential for the invasion of *A. actinomycetemcomitans* into immune cells^.^^55^ Therefore, to investigate whether β_2_ integrin is necessary for inflammasome activation during infection with *A. actinomycetemcomitans*, we focused on β_2_ integrin in macrophage-1 antigen (MAC-1, CR3, integrin α_M_β_2_) and it can also serve as a receptor for diverse microbial ligands.^56,57^ The caspase-1 activation, IL-1β and IL-6 release were reduced in BMDMs administered an antibody against CD11b, but not control IgG or an inhibitory antibody against CD18 during infection with *A. actinomycetemcomitans* ATCC29522 (Fig. 6a–c). Anti-CD11b antibody did not inhibit bacterial growth (Fig. S6a). We also assessed whether blocking CD11b affects inflammasome activation by other stimulants, nigericin, LPS-EB transfection, heat-killed Aa, or LPS from Aa. IL-1β release from BMDMs was not changed with or without anti-CD11b administration (Fig. 6d). Furthermore, we examined the effects of anti-CD11b on bacterial internalization. The infected macrophages were analyzed by immunostaining using anti-*A. actinomycetemcomitans* and anti-LAMP1 antibody. LAMP1 is a marker for late endosome and phagolysosome, and also useful for immunostaining to examine pathogen localization.^58–60^ Immunostaining showed that the co-localization of bacteria and LAMP1 diminished by the administration of anti-CD11b antibody in BMDMs infected with *A. actinomycetemcomitans* (Fig. 6e). Furthermore, we examined the binding ability of *A. actinomycetemcomitans* using recombinant α_M_β_2_ integrin (Mac-1, CD11b/CD18) and both immunostaining and flow cytometry*. A. actinomycetemcomitans* was not co-localized with α_M_β_2_ integrin, and the intensity of CD11b-FITC with *A. actinomycetemcomitan*s was not altered by the administration of α_M_β_2_ integrin (Fig. S6b, c). We next counted the number of internalized *A. actinomycetemcomitans* by immunostaining. The intracellular or cell-associated bacteria were decreased by the addition of anti-CD11b compared to control IgG (Fig. 6f). On the other hand, the number of extracellular bacteria was increased by anti-CD11b administration in the course of infection (Fig. 6g), indicating that anti-CD11b antibody inhibits bacterial internalization in BMDMs. These data suggest that CD11b is essential host factor for internalization of living *A. actinomycetemcomitans* but not functions by direct interaction with the bacteria.

### Arthritis and IL-1β secretion in paws of mice infected with *A. actinomycetemcomitans* are inhibited by PMB, chloroquine, and anti-CD11b antibody

**Fig. 7:**
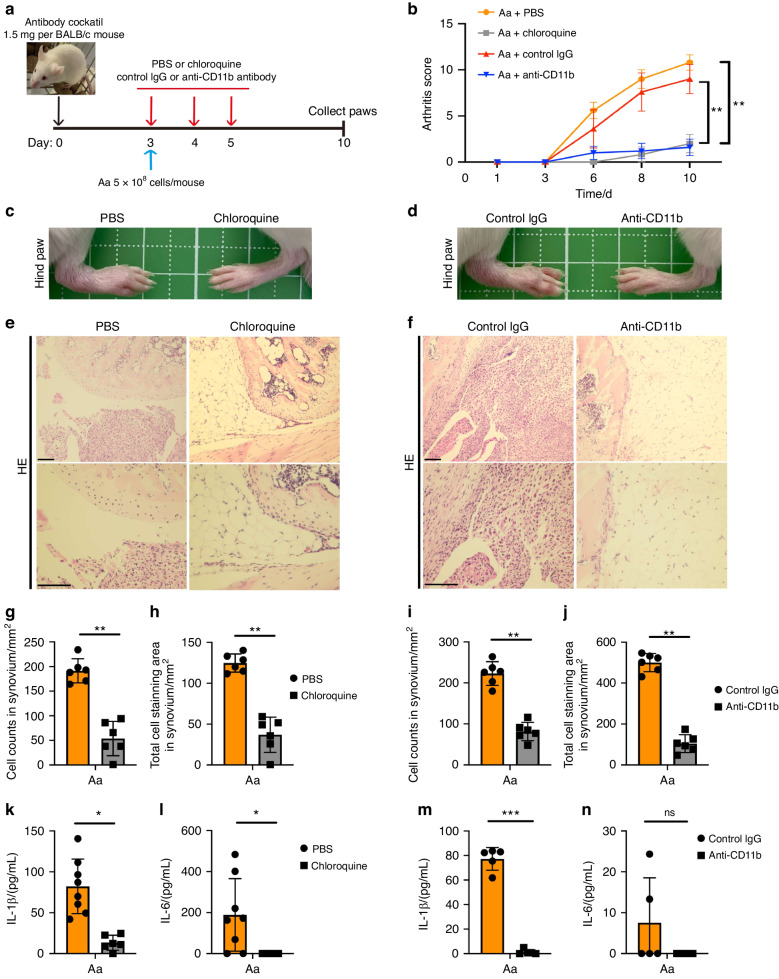
Inflammasome inhibition with chloroquine and anti-CD11b antibody attenuates arthritis in CAIA model. BALB/c mice were intraperitoneally injected with 1.5 mg of anti-collagen antibody on day 0, followed by the injection of chloroquine, anti-CD11b antibody, PBS or control IgG on days 3, 4, 5 and *A. actinomycetemcomitans* (Aa) on day 3. The arthritis scores were then monitored for 10 days. All paws were collected on day 10 for HE staining or cytokine analysis (PBS, *n* = 8; chloroquine, *n* = 6; control IgG, *n* = 5; anti-CD11b antibody, *n* = 5). **a** Schematic of CAIA model infected with *A. actinomycetemcomitans* (Aa) and administered 10 μL/g of liposomes containing clodronate or PBS. **b** Arthritis score. **c**, **d** Representative photographs of hind paws. **e**, **f** Representative HE staining of hind paws. Scale bar, 100 μm. **g**, **i** Cell number in 1 mm^2^ synovium. **h**, **j** Stained cell area in 1 mm^2^ synovium. **k**–**n** ELISA analysis for IL-1β and IL-6 release in paws. **g**–**n** Data are shown as the mean ± SD of triplicates and are representative of three independent experiments. ^∗^*P* < 0.05, ^∗∗^*P* < 0.01 and ^∗∗∗^*P* < 0.001 indicate a statistically significant difference using a *t*-test (ns not significant)

To analyze whether the inhibition of inflammasome activation during infection with *A. actinomycetemcomitans* is effective for preventing RA, we used PMB, chloroquine and anti-CD11b antibody as inhibitors. We established a CAIA model in which BALB/c mice in combination with infection of *A. actinomycetemcomitans* ATCC29522 and administered the above-mentioned inhibitors (Fig. 7a and S7a). Induced arthritis by intraperitoneal infection of *A. actinomycetemcomitans* in BALB/c mice was suppressed by the administration of chloroquine, anti-CD11b antibody, or PMB (Fig. 7b and S7b). Paw swelling was also attenuated in BALB/c mice treated by chloroquine, anti-CD11b antibody or PMB, compared with those administered PBS or control IgG (Fig. 7c, d and S7c). Furthermore, HE staining revealed a relatively small population of infiltrating cells in paws from BALB/c mice administered chloroquine or anti-CD11b antibody (Fig. 7e, f). We also counted cell number and measured cell staining area in 1 mm^2^ synovium. The administration of chloroquine or anti-CD11b antibody significantly reduced cell number and staining area in synovium (Fig. 7g-j).IL-1β secretion in paws was significantly decreased by the administration of chloroquine, anti-CD11b antibody, or PMB (Fig. 7k, m, and S7d). IL-6 secretion in paws was significantly decreased by the administration of chloroquine or PMB but not by the administration of anti-CD11b antibody (Fig. 7l, n and S7e). We also established a CAIA model using BALB/C mice administered LPS from *A. actinomycetemcomitans* (Fig. S7a). PMB diminished the arthritis score, cell swelling, and IL-1β and IL-6 secretion in paws as well as combined CAIA model of *A. actinomycetemcomitans* infection (Fig. S7b–d). To assess anti-CD11b antibody effects to immune cell migration in vivo, we examined CAIA model with using LPS from *E. coli* as a general booster for this model (Fig. S8a). Anti- CD11b treatment did not suppress paws swelling, arthritis severity, and cytokines production in paws compared to control IgG treatment (Fig. S8b–e). We also checked ratio of F4/80 positive cells in the spleen with anti-CD11b antibody or control IgG treatment. F4/80 is the major marker of mice macrophages. F4/80 positive cells ratio in the spleen was not altered with anti-CD11b antibody treatment compared with control IgG treatment (Fig. S8f). These data suggest that anti-CD11b antibody inhibits the arthritis severity with *A. actinomycetemcomitans* infection but not LPS EC treatment and did not suppress macrophages population in the spleen. Together, these data demonstrated that the inhibition of inflammasome activation in macrophages during infection with *A. actinomycetemcomitans* suppresses the induction of RA.

### Caspase-11 drives IL-1β secretion in paws and affects clinical severity of arthritis in mice infected with *A. actinomycetemcomitans*

**Fig. 8:**
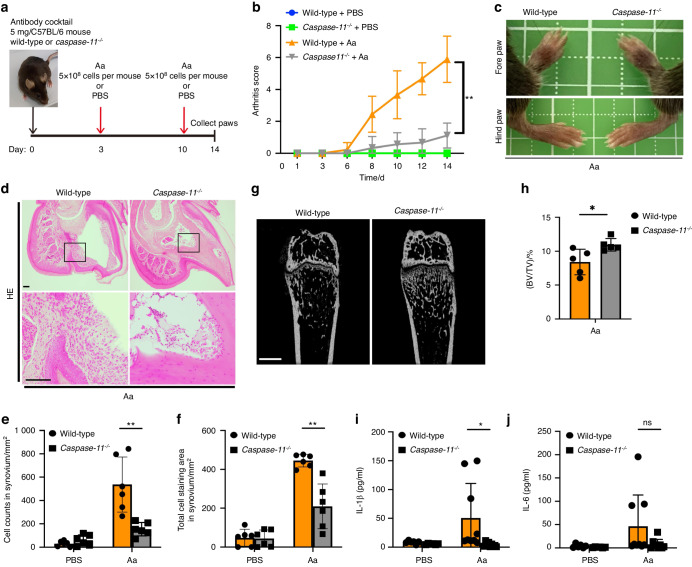
Caspase-11 drives arthritis via IL-1β secretion in CAIA model infected with *A*. *actinomycetemcomitans*. C57BL/6 wild-type or caspase-11-deficient mice were intraperitoneally injected with 5 mg of anti-collagen antibody on day 0 and with *A. actinomycetemcomitans* (Aa) or PBS on days 3 and 10. The arthritis scores were then monitored for 14 days. All paws were collected on day 14 for HE staining, μCT, or cytokine analysis (wild-type + PBS and *Caspase-11*^*−/−*^ + PBS, *n* = 6 mice per group, wild-type + Aa and *Caspase-11*^*−/−*^ + Aa, *n* = 9 mice per group). **a** Schematic of CAIA model infected with *A. actinomycetemcomitans* (Aa) using C57BL/6 wild-type or caspase-11-deficient mice. **b** Arthritis score. **c** Representative photographs of fore and hind paws. **d** Representative HE staining of fore and hind paws. Scale bar, 100 μm. **e** Cell number in 1 mm^2^ synovium. **f** Stained cell area in 1 mm^2^ synovium. **g** Representative two-dimensional μCT images of longitudinal sections of distal femur. Scale bar, 500 μm. **h** Bone volume per total tissue volume (BV/TV). **i**, **j** ELISA analysis for IL-1β and IL-6 release in paws. **b** ^∗∗^*P* < 0.01 indicates a statistically significant difference using a one-way ANOVA with the Tukey test. **e**–**j** Data are shown as the mean ± SD of triplicates and are representative of three independent experiments. ^∗^*P* < 0.05 and ^∗∗^*P* < 0.01 indicate a statistically significant difference using a *t*-test (ns not significant)

Finally, to further examine the relationship between inflammasome activation and the exacerbating of RA during infection with *A. actinomycetemcomitans*, we used caspase-11-deficient mice. Although C57BL/6 mice have lower sensitivity than BALB/c mice when used as a CAIA model, we could establish a CAIA model using C57BL/6 mice in combination with *A. actinomycetemcomitans* ATCC29522 infection (Fig. 8a). Joint swelling and immune cell infiltration were significantly lower in caspase-11-deficient mice than in wild-type mice after *A. actinomycetemcomitans* infection (Fig. 8b–d). We counted cell number and measured cell staining area in 1 mm^2^ synovium. Caspase-11 deficiency significantly reduced cell number and staining area in synovium (Fig. 8e, f). We also examined bone destruction in the hind paw using a μCT analysis. The cancellous bone density in wild-type mice was lower on μCT images compared with those of caspase-11-deficient mice (Fig. 8g). We also examined μCT analysis and found that bone volume per total tissue volume (BV/TV) increased significantly in cancellous bone in a caspase-11-dependent manner (Fig. 8h). Other markers, such as the trabecular number (Tb. N.), trabecular thickness (Tb. Th.), and trabecular separation (Tb. Sp.) did not differ significantly between wild-type and caspase-11-deficient mice (Data not shown) Furthermore, the IL-1β level was significantly reduced in paws from caspase-11-deficient mice after *A. actinomycetemcomitans* infection (Fig. 8i). The IL-6 levels in paws did not differ significantly between wild-type and caspase-11-deficient mice (Fig. 8j). These data suggest that inflammasome activation mediated by caspase-11 during infection with *A. actinomycetemcomitans* exacerbates arthritis following IL-1β upregulation and immune cell infiltration.

## Discussion

In this study, we demonstrated that intraperitoneal infection with a periodontitis-associated bacterium, *A. actinomycetemcomitans*, exacerbates arthritis in CAIA model by inducing cell infiltration into the inflamed synovium and diminution of bone volume by stimulating caspase-11-mediated IL-1β secretion from phagocytic cells. When the 129/Sv mouse strain, which lacks caspase-11 mRNA expression, was used as a modified CAIA model in the combination with *A. actinomycetemcomitans* infection, arthritis and IL-1β secretion in the paws were also not observed (data not shown). Together, these findings suggest that caspase-11 function in phagocytic cells is essential to exacerbate arthritis during infection with *A. actinomycetemcomitans*. Since we did not detect *A. actinomycetemcomitans* in the spleen and blood using an anti- *A. actinomycetemcomitans* antibody or PCR in oral infection (data not shown), we concluded that oral infection of *A. actinomycetemcomitans* did not induce the expansion of bacteria to the whole body in mice. We consider the mice’s oral microbiota difference from humans suppresses the bacterial colonization on oral tissue or the transfer to blood. In our research, we infected *A. actinomycetemcomitans* intraperitoneally to mimic human diseases directly.

Previous reports have shown that *A. actinomycetemcomitans* contributes to the induction of hypercitrullination, and the production of citrullinated RA autoantigens by LtxA promotes ACPAs generation.^15,61^ However, no reports have shown that *A. actinomycetemcomitans* infection aggravates RA after autoantigens presentation, and our data suggest that *A. actinomycetemcomitans* infection potentiates not only the facilitation of ACPAs generation, but also exacerbates RA after autoantigens presentation.^15,62^ Because CAIA model focuses on the step to trigger arthritis after pre-clinical stage in patients with RA containing 20-30% of ACPA negative ones, our findings support an understanding of the possible mechanisms for the induction of ACPAs-negative RA. On the other hand, infection with *P. gingivalis* did not exacerbate arthritis, and cell infiltration into the inflamed synovium was not observed in a CAIA model even though infection with *P. gingivalis* induces inflammasome activation in macrophages. It may be possible to be considered that *P. gingivalis* infection is important factor to exacerbate RA through progression of autoantigen presentation for ACPAs.^63,64^

We uncovered macrophages depletion by clodronate liposome suppress arthritis symptoms in a CAIA model with *A. actinomycetemcomitan* infection. However, many studies have investigated the relationship between macrophages and arthritis.^14,65^. Some previous studies have demonstrated that macrophages infiltrate synovium and myeloid-derived TNF controls severity in the CAIA model.^66,67^ Human arthritis patient data describe that IL-1β from M1 like macrophages in the synovium, has some effect on the progression of arthritis, and the neutralization of antibodies for IL-1β suppresses arthritis.^68,69^ IL-1β release by inflammasome activation from macrophages is essential in triggering or exacerbating arthritis symptoms. Since we do not know when Aa activates inflammasome in the patient macrophages, we cannot assume critical stage precisely. However, periodontitis associated bacteria can colonize long term on human periodontal pocket and possibly always activate inflammasome leading progression of RA. We used anti-Ly6G antibody to deplete neutrophils and clodronate liposome to deplete macrophages in preliminary CAIA experiments. As a result, clodronate liposome not anti-Ly6G antibody diminished arthritis severity with Aa infection, and we focused on macrophages.

We also showed that three inhibitors (PMB, chloroquine, and anti-CD11b antibody) of inflammasome activation in BMDM suppressed arthritis following cell infiltration into the inflamed synovium by attenuating IL-1β secretion in the paws of a CAIA model infected with *A. actinomycetemcomitans*. These data suggest that inflammasome activation in phagocytic cells, such as macrophages, plays a key role in the induction of arthritis progression in response to infection with *A. actinomycetemcomitans*. However, regarding anti-CD11b antibody treatment to mice possibly affect general function of macrophages,^70^ we cannot exclude the possibility that the macrophages are dysfunction by anti-CD11b antibody treatment while progressing inflammation in vivo. At least, this antibody suppressed arthritis by *A. actinomycetemcomitan* infection but not LPS EC treatment in our established model. Our results that the inhibitors depress inflammasome activation during infection with *A. actinomycetemcomitans* may suggest new pharmaceutical approaches to controlling arthritis severity.

Experimental caspase-11-mediated inflammasome activation generally requires the transfer of LPS to the cytosol with transfection, but LPS from *A. actinomycetemcomitans* can activate inflammasomes without transfection. We speculated that LPS from *A. actinomycetemcomitans* can be transferred directly by phagocytosis or another incorporation system, after which it is recognized by caspase-11. Regarding the structure of LPS from *A. actinomycetemcomitans*, a putative structure of Lipid A and polysaccharide (O-antigen) have been reported.^71,72^ Lipid A consists of 6 fatty acyl chains and 2 phosphatase groups with glucosamine disaccharide, while O-antigen consists of a large variety of sugar residues in many combinations and glycosidic linkages. A special structure capable of entry into cells has not been identified in any previous reports. Furthermore, many reports conclude that LPS from *A. actinomycetemcomitans* regulates the expression of proinflammatory cytokines, such as *Il1b*, *Il-8*, or *Tnfa*, and induces IL-1β secretion to polymorphonuclear leukocytes (PMN), macrophages, and dendritic cells, but not to gingival epithelial cells in periodontal lesions.^73–75^ Together, since the transfection of LPS is generally a necessary step to activate caspase-11-mediated inflammasomes in vitro, our findings and previous reports are not sufficient to elucidate the mechanism by which LPS from *A. actinomycetemcomitans* can invade cells without requiring the transfection. Induction of type I IFN via the TLR4 pathway is necessary to activate the caspase-11-mediated noncanonical inflammasome.^76^ However, IFNβ release from macrophages by LPS from *P. gingivalis* treatment was low compared to LPS from *E. coli* or *A. actinomycetemcomitans* treatment. IL-6 and TNFα release with LPS from *P. gingivalis* treatment to macrophages were also low concentration compared to other bacterial LPS. Taken together, less release of these cytokines leads to failure to trigger inflammasome activation in macrophages and CAIA model. Previous reports also analyzed LPS structure from *P. gingivalis* and the LPS longer carbon chains than in enterobacteria. They mentioned different acylation of lipid A compared to LPS from *E. coli*,^77^ and they speculated that this different structure leads to triggering different signal pathways such as the TLR2 pathway.^78^

We further examined the steps in the activation of inflammasomes in response to infection with *A. actinomycetemcomitans* from invasion into macrophages containing CD11b until cell death. Previous reports have shown that CD18 (β_2_ integrin) binds to LtxA from *A. actinomycetemcomitans* on neutrophils and enhances cytotoxic activity, but our data indicated that CD11b, rather than CD18, is important for entry into macrophages.^79,80^ However, we did not observe the binding of *A. actinomycetemcomitans* to CD11b in this study, and we speculate that the receptor facilitating *A. actinomycetemcomitans* invasion into macrophages is some other protein. Moreover, because examined three strains of *A. actinomycetemcomitans* including ATCC43717, which has low production of LtxA, activate caspase-11-mediated inflammasome, we suspect that LtxA is not an important factor for invasion.

We demonstrated that inflammasome activation by *A. actinomycetemcomitans* requires the degradation of LPS by lysosomal formation, but not by autophagosome formation with galectin-3 accumulation to lysosome. We hypothesized that LPS is exposed in the cytosol by a degradation system involving CD11b-dependent phagocytosis and lysosomal disruption, triggering its detection by caspase-11. However, previous reports have shown that *A. actinomycetemcomitans* induces autophagy, which contributes to the progression of cell death and bacterial internalization in human macrophages or junctional epithelium keratinocyte cells.^81,82^ Thus, the functions of autophagy remain poorly understood, and further analysis is needed to elucidate its importance during infection with *A. actinomycetemcomitans*.

In summary, we have firstly identified caspase-11-mediated inflammasome activation in macrophages possibility induces RA, *A. actinomycetemcomitans* activates caspase-11-mediated NLRP3 inflammasome, and discovered inhibitor for caspase-11-mediated inflammasome activation potentiate suppression of RA. However, because caspase-11 is specific gene in mice, it is necessary to consider the functional role of caspase-4/5 in human for ortholog of caspase-11 against *A. actinomycetemcomitans* infection. These findings may pave the way for advances in clinical treatments for RA induced by infection with *A. actinomycetemcomitans* or ACPAs-negative RA. Moreover, our suggestion to inhibit inflammasome activation possibly does not suppress the antigen presentation to induce RA, but we believe the inhibitors attenuate expansion of inflammation to joints, and result in recovering arthritis symptoms.

The conclusions in this study were based on results obtained using the CAIA model only, and a collagen-induced arthritis (CIA) model was not used, observing phagocytic cell effect not adaptive immune cell effect including antigen presentation. This study focuses on enhancing of arthritis severity by *A. actinomycetemcomitans* infection after injection of auto-antibody, we consider that bacterial infection progresses inflammation in joints indirectly by increasing inflammatory cytokine production in whole body but not induces auto-antigen presentation or triggers inflammation to joints directly. However, a recent study showed that infection with *A. actinomycetemcomitans* did not exacerbate macroscopic arthritis describing model for arthritis dependency between CIA and CAIA by *A. actinomycetemcomitans* infection.^83^ Future studies examining B cells and T cells are needed to uncover the mechanisms by which infection with *A. actinomycetemcomitans* induces RA fully. In the present study, we concluded that IL-1β is an important factor in the induction of RA by infection with *A. actinomycetemcomitans*, but our results obtained using a CAIA model indicate that IL-6 secretion in paws was also critical for the induction of RA by infection with *A. actinomycetemcomitans*. Generally, IL-6 is a critical factor in RA patients, and the effects of IL-6 on the CAIA model will need to be examined in the future. In addition, since previous reports demonstrated that NLRP3 inflammasome activation exacerbates arthritis.^20,84^ and IL-1β production contributes to the activation of immune cells, chondrocytes, osteoclasts, and induction of fibroblast proliferation,^65^ our findings must have some relevance to the enhancement of arthritis severity. However, these findings remain to be clinically validated in *A. actinomycetemcomitans* infected arthritis patients and we need further analysis with clinical samples. Furthermore, one previous paper demonstrated the linking of rheumatoid arthritis with *A. actinomycetemcomitans* infection.^15^ However, this link needed to be clarified in the mouse model. The mouse model for *A. actinomycetemcomitans* infection needs to be better established in the first place. In this study, we used a systemic infection model. We must establish an infection model of mice that is oral to the whole body. Moreover, we will determine whether or not to detect the protein citrullination in neutrophils by LtxA or antigen presentation to enhance arthritis by using a CAIA or CIA model in the future. In addition, we prepared proteins for ELISA or tissue for HE staining from BALB/c mice on day 10 such as previous studies,^66,85^ but this is shorter than general 15-day protocol. We could detect higher cytokines concentration or cell infiltration in the synovium compared to samples picked up on day 15. However, this short-term protocol possibly cannot evaluate chronic inflammation of arthritis. To understand mechanisms for progression of arthritis by Aa infection, we have to explore the best animal model for periodontitis with arthritis.

## Materials and Methods

### Ethics statement

All animal studies were performed in strict accordance with the Guidelines for Animal Experimentation of the Japanese Association for Laboratory Animal Science and the NIH guidelines. All protocols were approved by the Institutional Animal Care and Use Committee of Tokyo Medical and Dental University (approval number: A2019-131A). The experimental protocols covering the use of Living Modified Organisms, including bacterial mutants and gene-knockout mice, were approved by the Genetically Modified Organisms Safety Committee of Tokyo Medical and Dental University (approval number: G2018-021C5). The handling of the *A. actinomycetemcomitans*, *P. gingivalis*, and *P. intermedia* strains under biosafety level 2 conditions was approved by the Safety Control Committee for Pathogenic Microbes of Tokyo Medical and Dental University (approval number: M22019-004).

### Bacterial strains

*A. actinomycetemcomitans* (ATCC29522, ATCC43717, JP2, and JCM30399) were seeded on Trypticase soy agar (TSA) with 5% (v/v) sheep blood at 37 °C for 2 days under aerobic conditions. *P. gingivalis* (ATCC33277) and *P. intermedia* (ATCC25611) were seeded on CDC anaerobe blood agar at 37 °C for 5 days under anaerobic conditions (5% H_2_, 10% CO_2_, and 85% N_2_). Bacterial colonies were grown in BHI broth supplemented with 0.5% yeast extract, 0.1% L-cysteine, hemin (5 μg/mL), and vitamin K (1 μg/mL) under anaerobic conditions at 37 °C for 1 day. *E. coli* (MC1061) was grown in Luria-Bertani broth (1% tryptone, 0.5% yeast extract, 1% NaCl) under aerobic conditions at 37 °C for 1 day.

### Mice and preparation of macrophages

Wild-type C57BL/6 mice and BALB/c were purchased from Japan SLC (Tokyo, Japan). C57BL/6 background NLRP3-deficient, ASC-deficient, and caspase-11-deficient mice were housed in a pathogen-free facility. AIM2-deficient mice were provided by Dr. Kawaguchi (University of Tokyo). Caspase-11-deficient mice were provided by Masahiro Yamamoto (Osaka University). Myeloid cell-specific Atg5-deficient mice were generated by crossing the floxed Atg5 (RIKEN BioResource Research Center, Tsukuba, Japan) with the LysM- Cre transgenic mice (Jackson Laboratory). BMDMs were prepared from femurs and tibias of the above-mentioned mice and were cultured for 5–6 days in 10% FCS-RPMI 1640 supplemented with 30% mouse L-cell supernatant.

### Bacterial infection for immunoblotting

BMDMs were seeded at a density of 1.3 × 10^6^ cells per well in 6-well plates and infected with *A. actinomycetemcomitans* at an MOI of 50 per cell or administered an equivalent volume of the corresponding bacterial culture supernatant. At the indicated post-infection times, the cells were lysed with lysis buffer (25 mM Tris-HCl [pH7.4], 150 mmol/L NaCl, 1% NP40, complete protease inhibitor cocktail [Roche Diagnostics]), and the culture supernatants were precipitated by the addition of 10% trichloroacetic acid. The samples were loaded onto 15% or 10% SDS-PAGE, and caspase-1, IL-1β, GSDMD, caspase-11 or β-actin were detected using anti-caspase-1 (adipogen, Cat. Casper-1), anti-IL-1β (R&D, AF-401-NA), anti-GSDMD (abcam, Cat. ab180673), anti-caspase-11 (abcam, Cat. ab209845), and anti-β-actin (Merck, Cat. MAB1501) antibody, respectively. Nigericin (Sigma, Cat. N7143) and LPS EB (Invivogen, Cat. tlrl-3pelps) with FuGENE® HD Transfection Reagent (Promega, Cat. E2311) for transfection are used for positive control as NLRP3 inflammasomne activation.

### Bacterial infection for ELISA

BMDMs were seeded at a density of 3.5 × 10^5^ cells per well in 24-well plates and infected with *A. actinomycetemcomitans* at an MOI of 50 per cell or administered an equivalent volume of the corresponding bacterial culture supernatant, heat-inactivated bacteria, heat-killed bacteria, or culture supernatant from heat-killed bacteria. At the indicated post-infection times, the culture supernatants were collected and the released cytokines were quantified using ELISA (Invitrogrn: Cat. 88-7013-22 for IL-1β, Invitrogrn: Cat. BMS603-2 for IL-6, R&D: Cat. 42400-1 for IFNβ).

### LDH release assay

BMDMs were seeded at a density of 3.5 × 10^5^ cells per well in 24-well plates and infected with *A. actinomycetemcomitans* at an MOI of 50 per cell. At the indicated post-infection times, the culture supernatants were collected, and the lactate dehydrogenase (LDH) activity in the culture supernatants was measured using a CytoTox 96 assay kit (Promega, Madison, WI, United States, Cat. G178), in accordance with the manufacturer’s protocol.

### Bacterial counting with immunofluorescence

BMDMs were seeded at a density of 6.5 × 10^5^ cells per well in 6-well plates and infected with *A. actinomycetemcomitans* at an MOI of 1 per cell at the indicated post-infection times. The BMDMs were then administered 200 μg/mL of gentamycin for 1 h. The infected BMDMs were fixed, immunostained with anti-*A. actinomycetemcomitans* produced in this study and CD107a (LAMP-1) Monoclonal Antibody (eBio1D4B (1D4B) Cat. 14-1071-82), eBioscience^TM^ as described previous procedure,^86^ and then analyzed using a laser-scanning microscope (LSM-800; Carl Zeiss).

### LPS extraction from bacteria

LPS from *A. actinomycetemcomitans*, *Porphyromonas gingivalis*, *Prevotella intermedia*, and *E. coli* were extracted using an LPS extraction kit (Labotaq). Briefly, bacterial cultures were collected and lysed with phenol containing buffer and washed with 70% ethanol; LPS was then lysed with Tris-HCl buffer and boiled.

### Detection of lysosomal disruption with *A. actinomycetemcomitans* infection by immunostaining

BMDMs were seeded at a density of 6.5 × 10^5^ cells per well in 6-well plates and infected with *A. actinomycetemcomitans* at an MOI of 25 per cell at the indicated post-infection times or 1 mM of LLOMe for 2 h. The infected BMDMs were fixed, immunostained with anti-galectin-3 (SC-23938) and CD107a (LAMP-1) Monoclonal Antibody (eBio1D4B (1D4B) Cat. 14-1071-82), and then analyzed using a laser-scanning microscope (LSM-800; Carl Zeiss).

### CD11b blockade for BMDMs

BMDMs were infected in the presence of either 2.5, 5, or 10 μg/mL of an isotype control (Clone A95-1; BD Biosciences Cat. 553986), an inhibitory monoclonal antibody against CD11b (clone M1/70; BD Biosciences Cat. 553308) or CD18 (Clone M18/2; BD Biosciences Cat. 557437).

### Bacterial counting on blood agar plates

*A. actinomycetemcomitans* ATCC29522 was grown in BHI broth supplemented with 0.5% yeast extract, hemin (5 μg/mL), and vitamin K (1 μg/mL) under anaerobic conditions (5% H_2_, 10% CO_2_, and 85% N_2_) at 37 °C for 1 day. Cultures were normalized to OD_600_ 0.05 and incubated with PMB (Sigma, P4937), chloroquine (Sigma, C6628), or anti-CD11b antibody for 6 h in BHI broth supplemented with yeast extract, hemin, and vitamin K under anaerobic condition. Bacterial culture with inhibitors was incubated on TSA with 5% (v/v) sheep blood at 37 °C for 48 h. The colonies growing on the TSA were then counted.

### Bacterial counting in culture supernatants of BMDMs with CD11b blocked

BMDMs were seeded at a density of 1.3 × 10^6^ cells per well in 6-well plates and infected with *A. actinomycetemcomitans* at an MOI of 25 per cell with or without anti-CD11b antibody. At the indicated time, culture supernatants were collected and incubated on TSA with 5% (v/v) sheep blood at 37 °C for 48 h. The colonies growing on the TSA were then counted.

### CD11b binding with *A. actinomycetemcomitans* by immunostaining

Cultured *A. actinomycetemcomitans* ATCC29522 was mixed with mouse recombinant integrin alpha M beta 2 protein (R&D Cat.7959-AM-050) and incubated at 37 °C for 1 h. Mixtures were collected and incubated with anti-CD11b antibody conjugated FITC (eBioscience™ Cat.11-0112-41), anti-*A. actinomycetemcomitans* (Purified in this study), and anti-mouse antibody conjugated Cy3 for 1 h. Bacteria that were colocalized with CD11b were analyzed using a laser-scanning microscope (LSM-800; Carl Zeiss).

### Flowcytometry

Cultured *A. actinomycetemcomitans* ATCC29522 was mixed with mouse recombinant integrin alpha M beta 2 protein and incubated at 37 °C for 1 h. Mixtures were collected and incubated with anti-CD11b antibody conjugated FITC for 1 h. The collected samples were analyzed using a flow cytometer (EC800; SONY).

### Detection of low internal pH lysoeome

BMDMs were seeded at a density of 6.5 × 10^5^ cells per well in 6-well plates and infected with *A. actinomycetemcomitans* at an MOI of 50 per cell for 2 h. The BMDMs were then administered 200 μg/mL of gentamycin for 1 h. The infected BMDMs were fixed, stained with Lysotracker green DND26 (Thermo) and DAPI for 30 min, and then analyzed using a laser-scanning microscope (LSM-800; Carl Zeiss).

### Induction of CAIA

BALB/c, C57BL/6 wild-type, or caspase-11-deficient male mice between 8 and 12 weeks old were given 1.5 (150 μL) or 5 mg (500 μL) of Arthritogenic-CIA 5-Clone Cocktail (Chondrex) by intraperitoneal injection on day 0, followed by infection with *A. actinomycetemcomitans* ATCC29522 (5 × 10^8^ cells per mouse in 100 μL PBS)*, P. gingivalis* ATCC33277 (5 × 10^8^ cells per mouse in 100 μL PBS), or PBS or LPS from E. coli (LPS EC), or LPS from *A. actinomycetemcomitans* ATCC29522 (50 μg per mouse in 100 μL Tris-HCl) by intraperitoneal injection on day 3. For the C57BL/6 mice, *A. actinomycetemcomitans* ATCC29522 (5 × 10^8^ cells per mouse in 100 μL PBS) was injected intraperitoneally once again on day 10. Arthritis was scored on days 0 to 10 as previous reports.^87,88^: 0, no inflammation of joints in paw; 1, one joint with inflammation in the paw involving the digits; 2, 2 joints with inflammation in the paw involving the digits; 3, all joints with inflammation involving the digits; 4, all joints with inflammation involving the digits and severe inflammation of the entire paw resulting in ankylosis. The BALB/c mice were monitored until day 10, and the C57BL/6 mice were monitored until day 14.

### Administration of liposomes containing clodronate in the CAIA model

Phagocytic cells were depleted by the intraperitoneal injection of 10 μL/g of clodronate liposomes (Liposoma B.V., Netherlands) to mice on day 2 in the CAIA model. Control liposomes were intraperitoneally administered to control mice.

### Administration of PMB, chloroquine, or anti-CD11b in CAIA model

Mice in the CAIA model were intraperitoneally administered 80 mg/kg of PMB in 100 μL PBS, 160 mg/kg of chloroquine in 100 μL PBS, or 1.25 mg/kg of inhibitory monoclonal antibody against CD11b (clone M1/70) in 100 μL PBS. 100 μL PBS or an isotype control of IgG were intraperitoneally administered to control mice.

### Histology

The metatarsal and costal cartilages were collected from CAIA mice on days 10 or 14. The samples were fixed in 4% paraformaldehyde overnight and decalcified in EDTA, then paraffin-embedded and sectioned (3 μm). Hematoxylin and eosin Y (H&E) staining was performed using the standard protocol to visualize the infiltration of inflammatory cells into the joint. Cell counting and staining area in 1 mm^2^ were calculated by ImageJ software (Wright Cell Imaging Facility, Toronto Western Research Institute).

### μCT analysis

For the two-dimensional μCT analyses, the left femurs were fixed in 70% ethanol for 1 week. μCT scanning was performed using a Scan Xmate-L090H (Comscantecno). The two-dimensional structural indices were calculated using TRI/3D-BON software (RATOC System Engineering).

### ELISA analysis for CAIA model

All the paws were collected and incubated in liquid nitrogen. Cold paws were destroyed using SK mill (TOKKEN, Inc.), and the tissues were added to homogenized buffer (150 mmol/L NaCl, 10 mmol/L Tris-HCl [pH7.4], 5 mmol/L EDTA, 0.1% Nonidet P-40, 5% glycerol). All the fractions were centrifuged at 1 900 x *g* in a microcentrifuge at 4 °C. The supernatants were collected and centrifuged at 13 000 r/min at 4 °C. The supernatants were used as samples in ELISA analyses of IL-1β and IL-6.

### F4/80 positive cell counting

BALB/c mice spleens were collected and counted total cell number with a hemocytometer. F4/80 positive cells were collected by EasySep Mouse F4/80 Positive Selection Kit (Stem Cell Technology Cat.100-0659). Collected cells were counted with a hemocytometer and calculated ratio of F4/80 positive cells per total cells.

### Statistical analysis

All the data are presented as the mean ± SD of at least three determinations per experimental condition. All the experiments were performed at least three times, and representative results are shown in the Figs. Statistical analyses were performed using unpaired two-tailed Student *t*-tests or a one-way ANOVA followed by the Tukey test. Differences were considered significant at a *p*-value of < 0.05, 0.01, or 0.001.

### Supplementary information

Supplementary legends and figures
