# Supplementary material for: Caspase-11 mediated inflammasome activation in macrophages by systemic infection of A. actinomycetemcomitans exacerbates arthritis
Source: Int J Oral Sci. 2024 Aug 15;16:54. doi: 10.1038/s41368-024-00315-x (PMC11324795; doi:10.1038/s41368-024-00315-x)
Supplement: Supplementary file 1 — Supplementary legends and figures [file 41368_2024_315_MOESM1_ESM.pdf]

Supplementary figure legends

Fig. S1. ***A. actinomycetemcomitans*** **exacerbates arthritis in a CAIA model and activates inflammasomes in an AIM2-independent manner.**

a-e BALB/c mice were intraperitoneally injected with 1.5 mg of anti-collagen antibody on day 0, followed by the injection of liposomes containing clodronate or PBS on day 2 and LPS EC 10 μg/mouse on day 3. The arthritis scores were monitored for 10 days. The paws of all the mice were collected on day 10 for cytokine analysis (clodronate liposome, n=5; control liposome, n=7). **a** Schematic of CAIA model injected with LPS EC and administered 10 μL/g of liposomes containing clodronate or PBS. **b** Representative photographs of hind paws. **c** Arthritis score. **d** and **e** ELISA analysis showing the release of IL-1β and IL-6 in paws. (**c**, **d** and **e**) $^{*}P$ <0.05 and $^{**}P$ <0.01 indicate a statistically significant difference using a *t*-test (ns, not significant).

Fig. S2 AIM2 is not essential for inflammasome activation during infection with ***A. actinomycetemcomitans.***

BMDMs from wild-type or AIM2-deficient (*Aim2*$^{-/-}$) mice were infected with *A. actinomycetemcomitans* LtxA (+) (ATCC29522), LtxA (-) (ATCC43717), or LtxA (++) (JP2). Nigericin (5 μM) or Poly (dA:dT) (2 μg) were administered to 200 ng of LPS-

Supplementary figure legends

Fig. S1. ***A. actinomycetemcomitans*** **exacerbates arthritis in a CAIA model and activates inflammasomes in an AIM2-independent manner.**

a-e BALB/c mice were intraperitoneally injected with 1.5 mg of anti-collagen antibody on day 0, followed by the injection of liposomes containing clodronate or PBS on day 2 and LPS EC 10 μg/mouse on day 3. The arthritis scores were monitored for 10 days. The paws of all the mice were collected on day 10 for cytokine analysis (clodronate liposome, n=5; control liposome, n=7). **a** Schematic of CAIA model injected with LPS EC and administered 10 μL/g of liposomes containing clodronate or PBS. **b** Representative photographs of hind paws. **c** Arthritis score. **d** and **e** ELISA analysis showing the release of IL-1β and IL-6 in paws. (**c**, **d** and **e**) $^{*}P<0.05$ and $^{**}P<0.01$ indicate a statistically significant difference using a *t*-test (ns, not significant).

Fig. S2 AIM2 is not essential for inflammasome activation during infection with ***A. actinomycetemcomitans.***

BMDMs from wild-type or AIM2-deficient (*Aim2*$^{-/-}$) mice were infected with *A. actinomycetemcomitans* LtxA (+) (ATCC29522), LtxA (-) (ATCC43717), or LtxA (++) (JP2). Nigericin (5 μM) or Poly (dA:dT) (2 μg) were administered to 200 ng of LPS-

EB-primed BMDMs as a control to activate NLRP3 or AIM2 inflammasomes. Brain heart infusion (BHI) media was added as a negative control for inflammasome activation. **a** Immunoblot analysis for pro-form of caspase-1 (procaspase-1, 45 kDa), pro-form of IL-1β (pro-IL-1β, 31kDa), and β-actin in cells and the subunit of the active form of caspase-1 P20 (active caspase-1, 20 kDa) and the mature form of IL-1β (mature IL-1β, 17 kDa) in supernatants. **b** ELISA analysis for IL-1β release in supernatants. (**a**) Blots are representative of three independent experiments. (**b**) Data are shown as the mean±SD of triplicates and are representative of three independent experiments.

Fig. S3 Clinical isolated strain of *A. actinomycetemcomitans* induces cell death and IL-1β release in a caspase-11 partially dependent manner.

BMDMs from wild-type or *Caspase-11*$^{-/-}$ mice were infected with *A. actinomycetemcomitans* JCM30399 for 12 hours. **a** LDH release in supernatants. **b** ELISA analysis for IL-1β release in supernatants. **P* <0.05 and ***P* <0.01 indicate a statistically significant difference using a *t*-test.

Fig. S4 PMB has no effect to bacterial growth and LPS from *A. actinomycetemcomitans* induces proinflammatory cytokines release.

a *A. actinomycetemcomitans* was cultured with 5 μg/mL of PMB in RPMI1640 medium for 12 hours. Colony forming units were counted on TSA blood agar plates. **b-d** BMDMs were incubated with LPS from *E. coli* MC1061 (LPS Ec), *P. gingivalis* ATCC33277 (LPS Pg), *P. intermedia* ATCC25611 (LPS Pi), or *A. actinomycetemcomitans* ATCC29522 (LPS Aa). Supernatants were harvested at 2, 4, or 6 hours post-incubation. ELISA analysis for IL-6, TNFα, and IFNβ release in supernatants. (**b-d**) Data are shown as the mean±SD of triplicates and are representative of three independent experiments.

Fig. S5 Effect of chloroquine administration on inflammasome activation and bacterial growth

a and **b** BMDMs were infected with *A. actinomycetemcomitans* ATCC29522 (Aa) or added BHI media for 4 hours. **a** Representative staining analysis for internal low pH of lysosome with lysotracker green reagent. Scale bar, 50 μm. **b** Images of fluorescene intensity analyzed by laser-scanning microscope. **c** BMDMs were administered LPS-EB or nigericin for 30 minutes, heat-killed Aa or LPS Aa for 9 hours, or transfected with

LPS-EB for 10 hours. ELISA analysis for IL-1β release in supernatants. **d** *A. actinomycetemcomitans* was cultured with 100 μg/mL of chloroquine in RPMI1640 medium for 12 hours. Colony forming units counted on TSA blood agar plates.

(A, B, and D) Data are shown as the mean±SD of triplicates and are representative of three independent experiments.

Fig. S6 CD11b does not bind and colocalize with *A. actinomycetemcomitans*.

a *A. actinomycetemcomitans* was cultured with 10 μg/mL of anti-CD11b antibody or isotype control IgG in RPMI1640 medium for 12 hours. Colony forming units were counted on TSA blood agar plates. **b** and **c** *A. actinomycetemcomitans* was co-incubated with recombinant $\alpha_M\beta_2$ integrin or PBS for 1 hour. **b** Representative immunostaining analysis for colocalization of *A. actinomycetemcomitans* with CD11b. Scale bar, 10 μm. CD11b was stained using FITC and *A. actinomycetemcomitans* ATCC29522 was stained using Cy3. **c** Flow cytometry analysis of CD11b binding to *A. actinomycetemcomitans.* (**a**) Data are shown as the mean±SD of triplicates and are representative of three independent experiments. (**b** and **c**) Images and histograms are representative of three independent experiments.

Fig. S7 Inhibition of inflammasome activation by infection with *A. actinomycetemcomitans* or LPS from *A. actinomycetemcomitans* with PMB suppresses arthritis in CAIA model.

BALB/c mice were intraperitoneally injected with 1.5 mg of anti-collagen antibody on day 0, followed by injection with PMB or PBS on days 3, 4, 5, and *A. actinomycetemcomitans* or LPS from *A. actinomycetemcomitans* on day 3. The arthritis scores were monitored for 10 days. All paws were collected on day 10 for HE cytokines analysis (n=3 mice per group). **a** Schematic of CAIA model infected with *A. actinomycetemcomitans* infection and administered 10 μL/g of liposomes containing clodronate or PBS. **b** Arthritis score. **c** Representative photographs of forepaw. **d** and **e** ELISA analysis for IL-1β and IL-6 release in paws. (**d** and **e**) Data are shown as the mean±SD of triplicates and are representative of three independent experiments. ***P* <0.01 indicates a statistically significant difference using a *t*-test.

Fig. S8 anti-CD11b antibody treatment does not affect progression of arthritis in CAIA model with using LPS and population of F4/80 positive cells in the spleen.

a-e BALB/c mice were intraperitoneally injected with 1.5 mg of anti-collagen antibody on day 0, followed by the injection of anti-CD11b antibody or control IgG on days 3, 4,

5 and LPS EC 10 μg/mouse on day 3. The arthritis scores were then monitored for 10 days. All paws were collected on day 10 for cytokine analysis (control IgG, n=4; anti-CD11b antibody, n=4). **a** Schematic of CAIA model infected with LPS EC injection and administered anti-CD11b antibody or control IgG. **b** Arthritis score. **c** Representative photographs of fore and hind paws. **d** and **e** ELISA analysis for IL-1β and IL-6 release in paws. **f** BALB/c mice were intraperitoneally injected with anti-CD11b antibody or control IgG on day 0. Spleens were collected and extracted F4/80 positive cells. Percentage of F4/80 positive cells number. (**b, d**, **e** and **f)** Data are shown as the mean±SD of triplicates and are representative of three independent experiments. (ns, not significant).

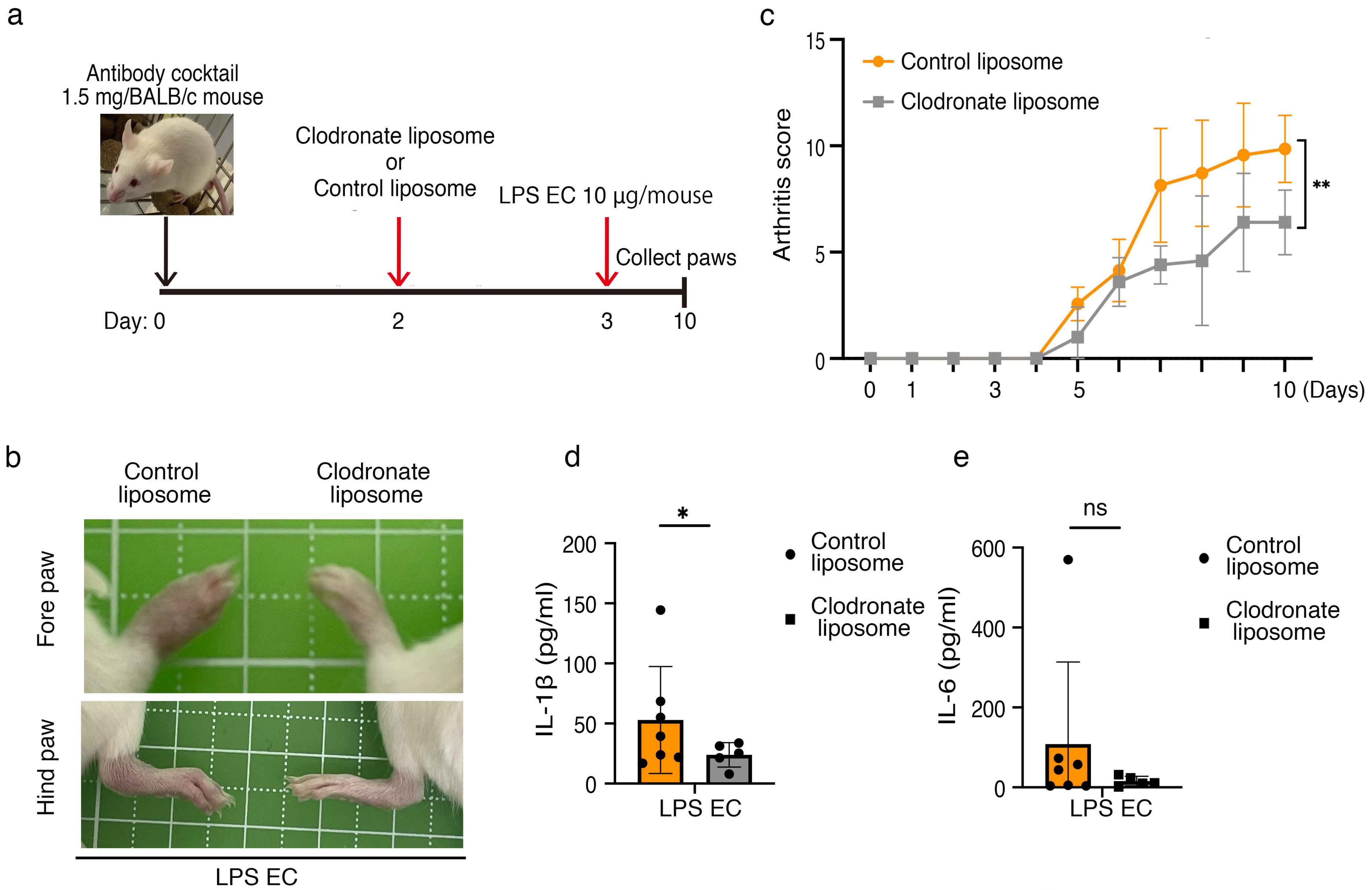

Okano et al. Fig S1

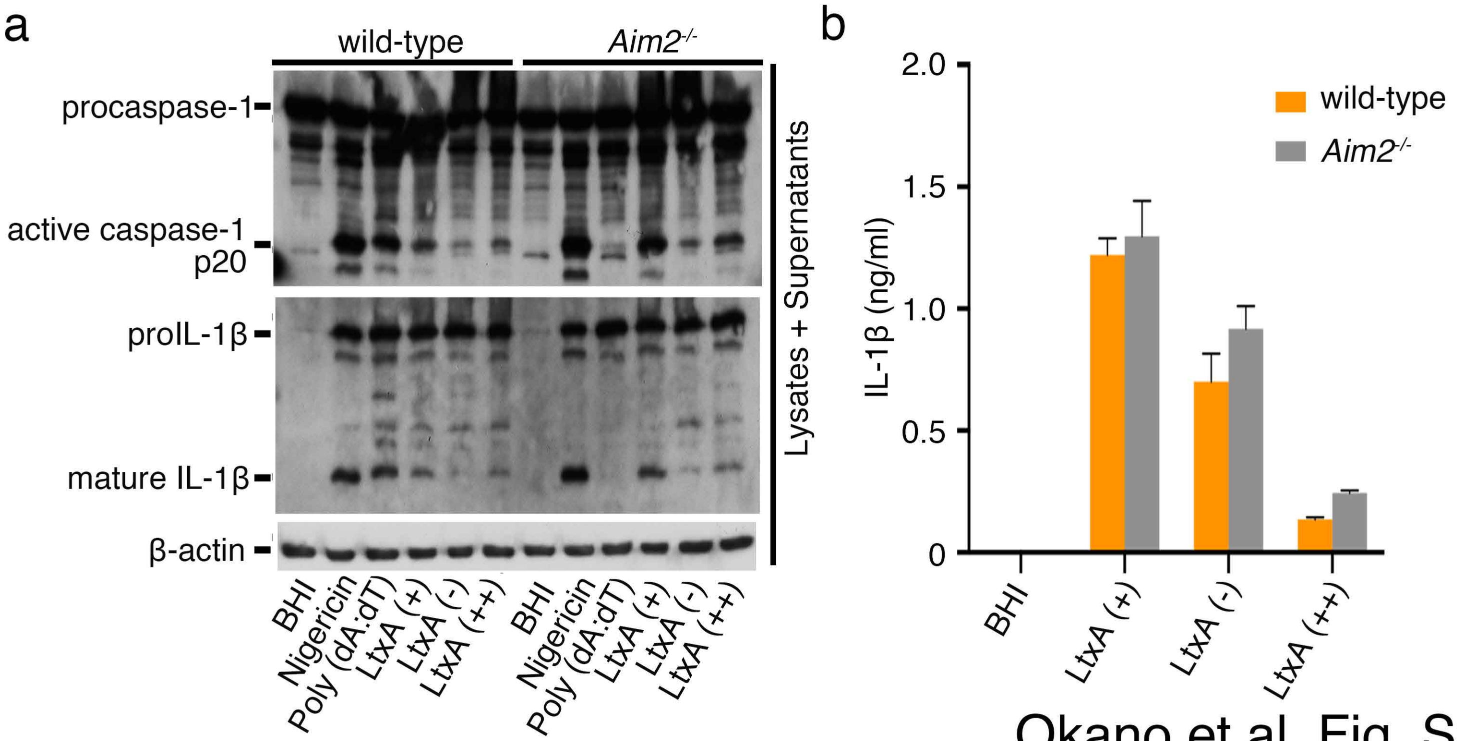

Okano et al. Fig. S2

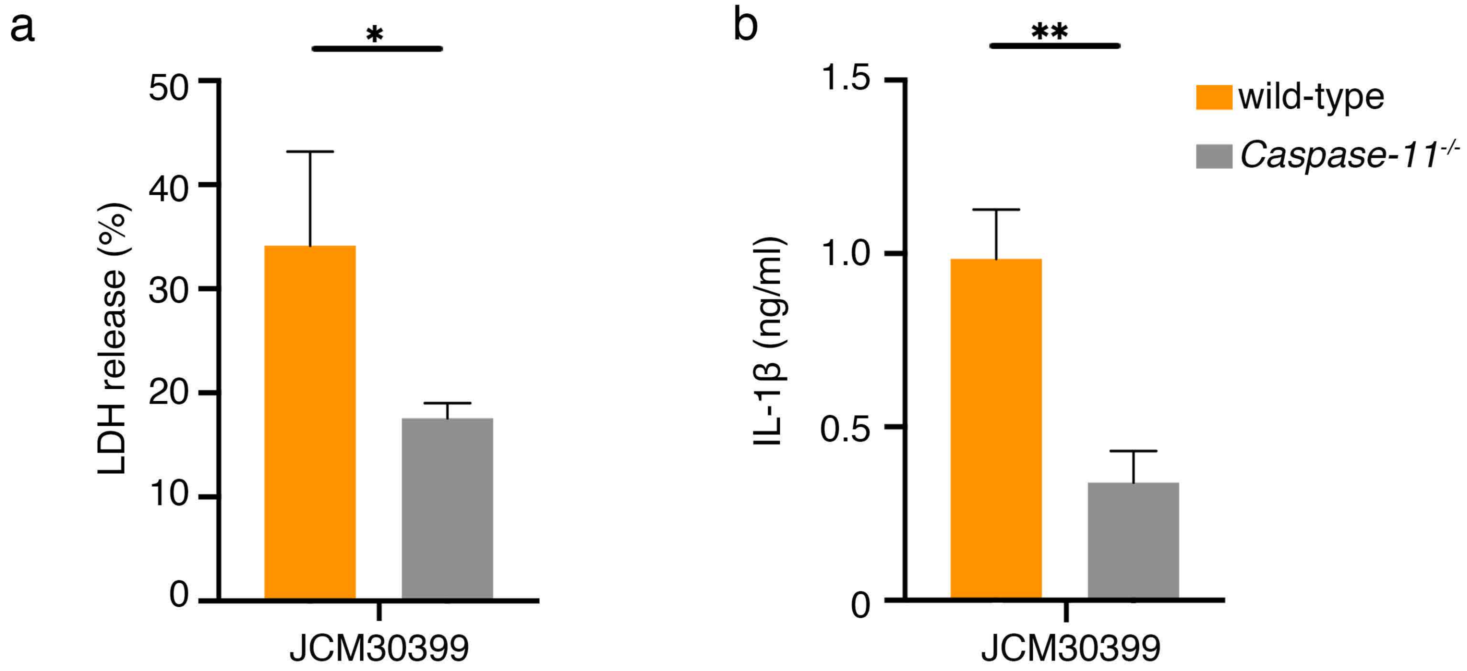

Okano et al. Fig. S3

Okano et al. Fig. S4

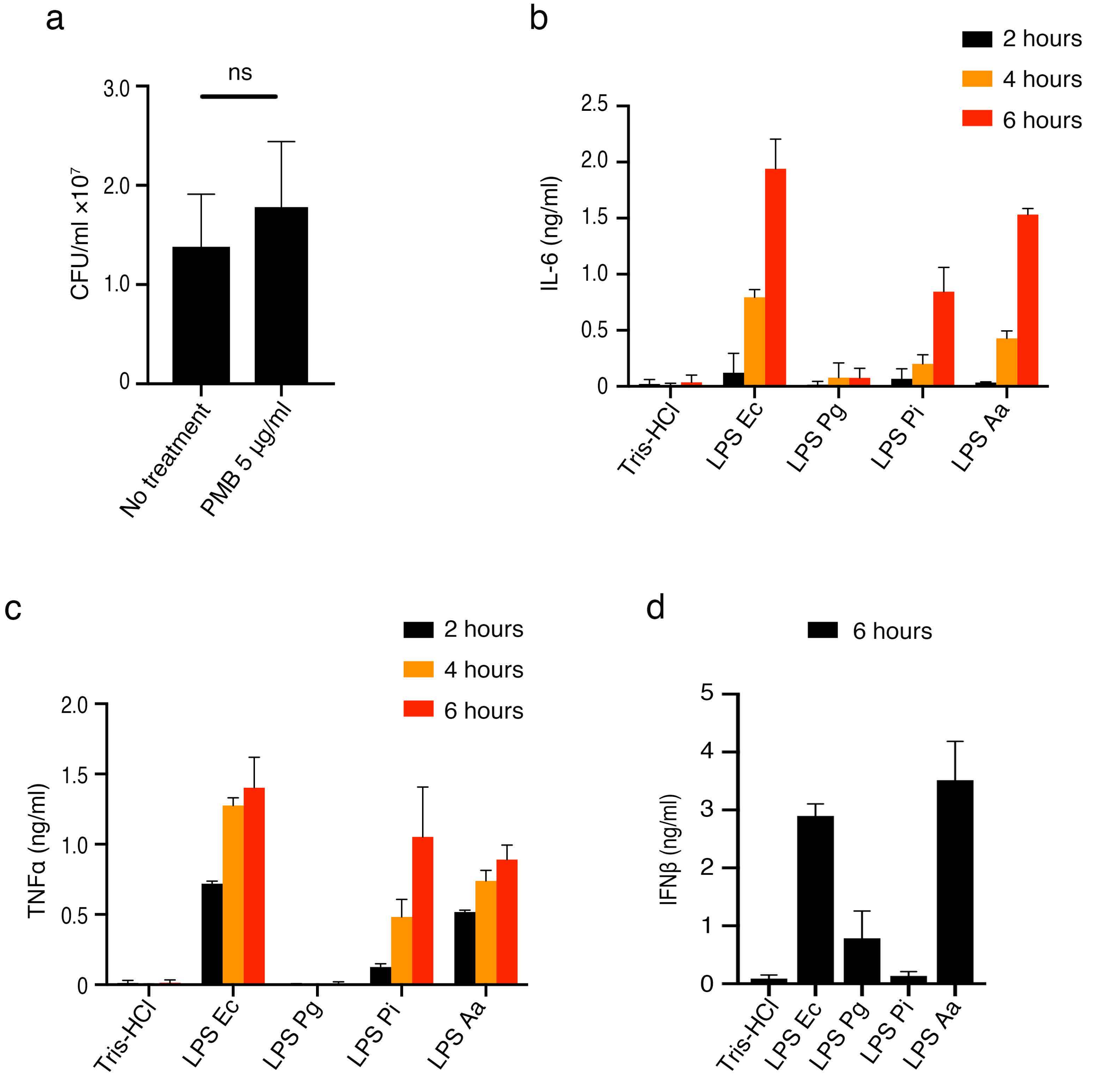

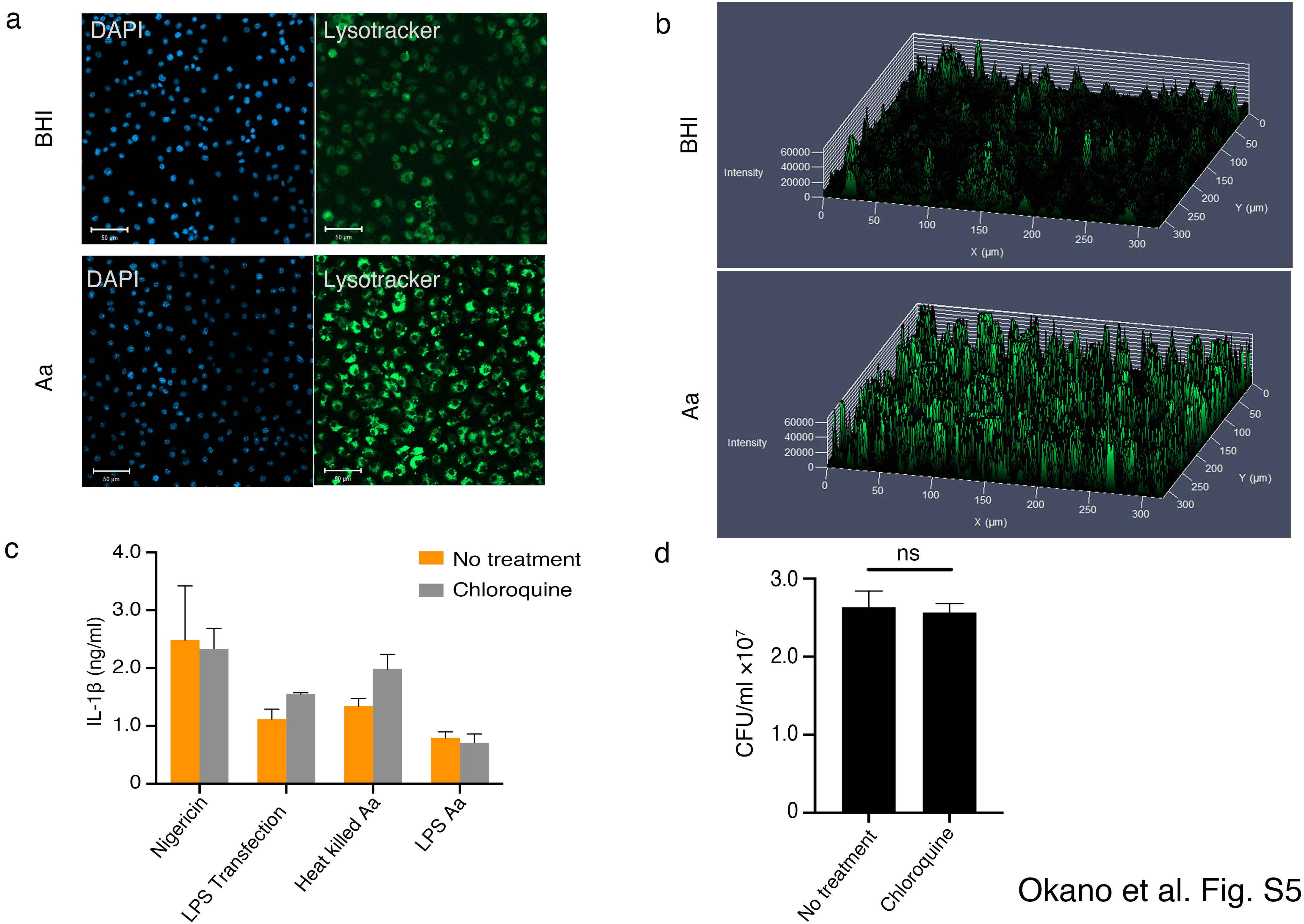

Okano et al. Fig. S5

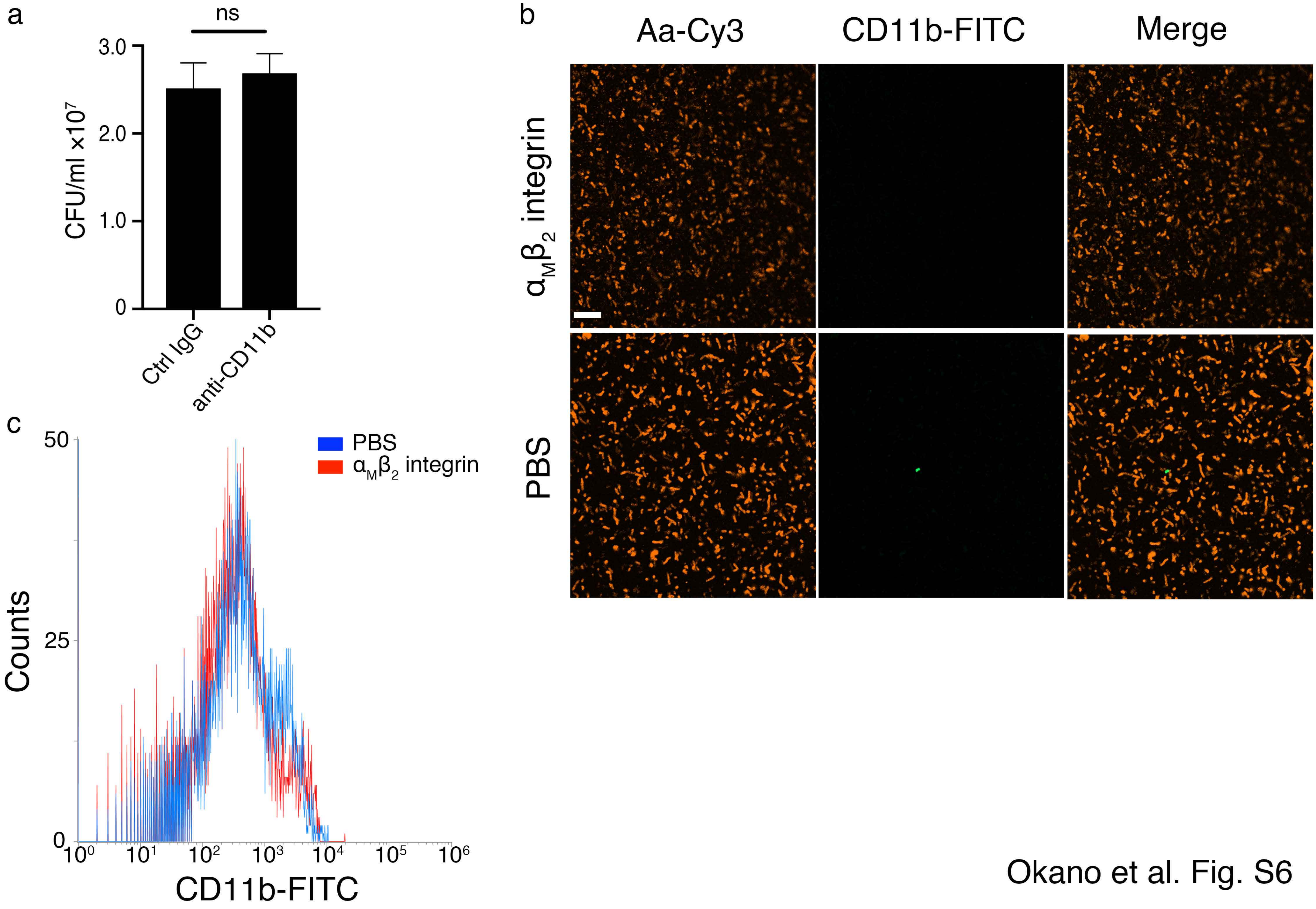

Okano et al. Fig. S6

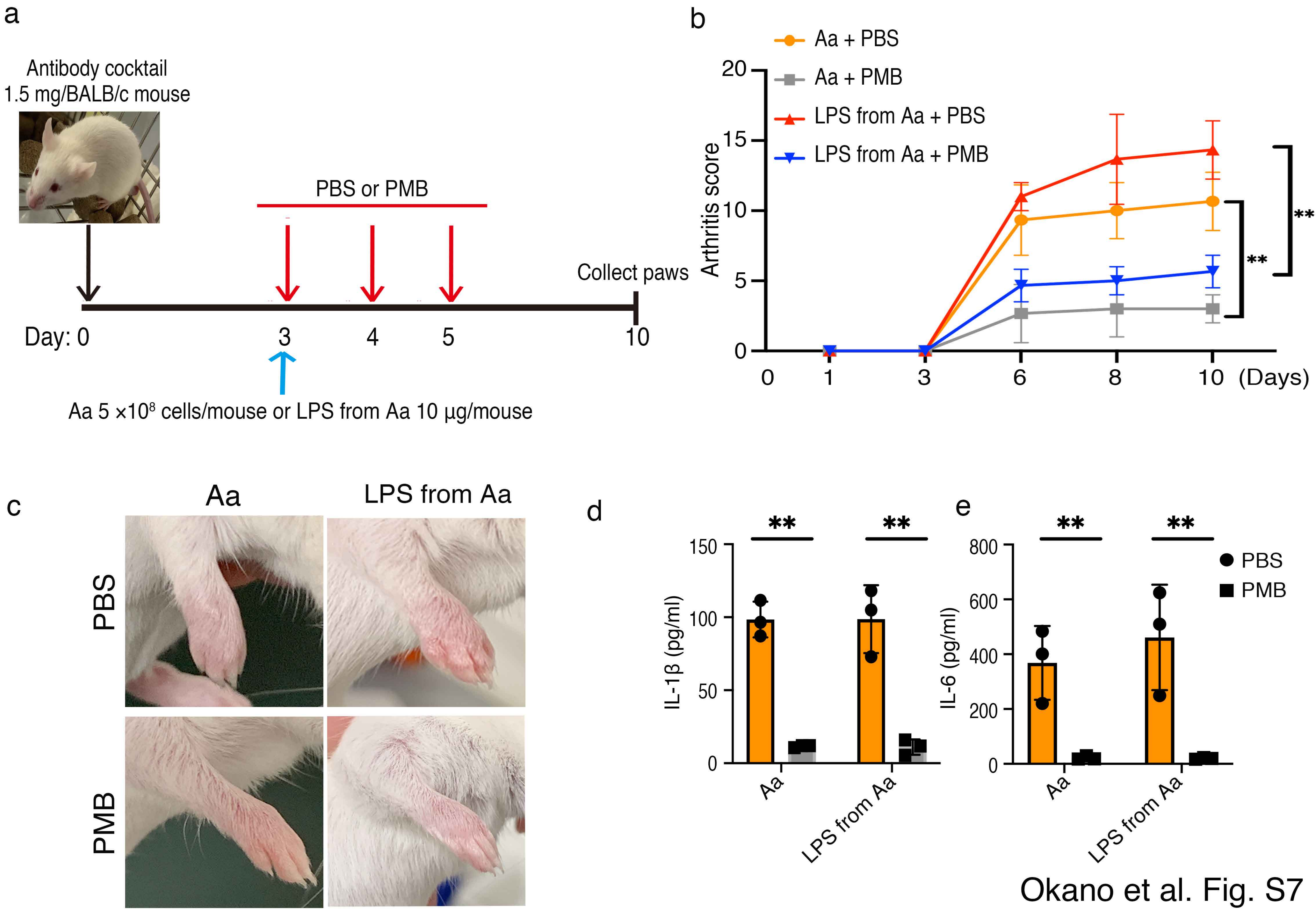

Okano et al. Fig. S7

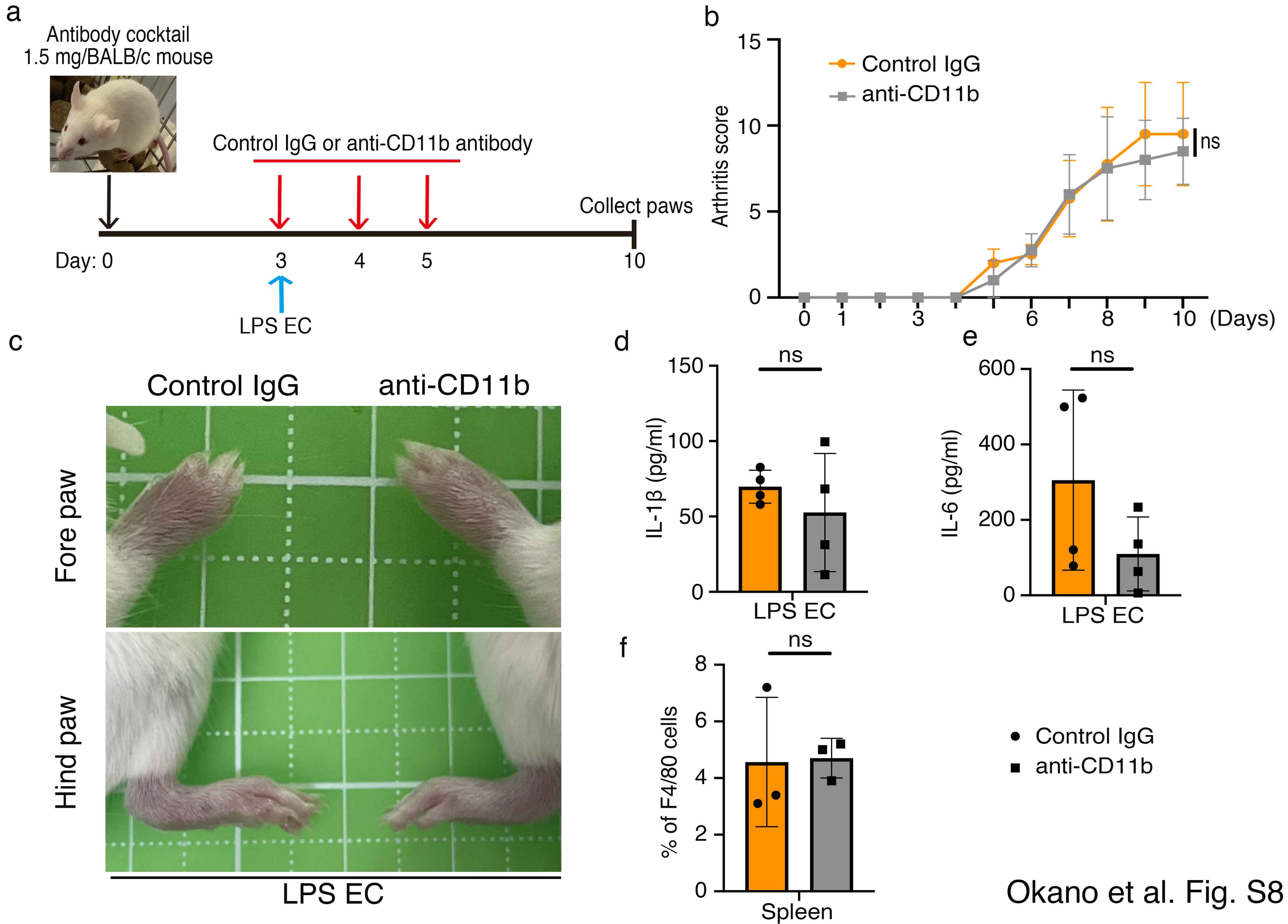

Okano et al. Fig. S8
